# Nutraceuticals for Complementary Treatment of Multisystem Inflammatory Syndrome in Children: A Perspective from Their Use in COVID-19

**DOI:** 10.3390/life12101652

**Published:** 2022-10-20

**Authors:** Diego Estrada-Luna, Elizabeth Carreón-Torres, Susana González-Reyes, María Fernanda Martínez-Salazar, María Araceli Ortiz-Rodríguez, Esther Ramírez-Moreno, José Arias-Rico, Angélica Saraí Jiménez-Osorio

**Affiliations:** 1 Área Académica de Enfermería, Instituto de Ciencias de la Salud, Universidad Autónoma del Estado Hidalgo, Circuito Ex Hacienda La Concepción S/N, Carretera Pachuca-Actopan, San Agustín Tlaxiaca 42160, Mexico; 2 Department of Molecular Biology, Instituto Nacional de Cardiología Ignacio Chávez, Juan Badiano 1, Sección XVI, Tlalpan, Mexico City 14080, Mexico; 3 Facultad de Medicina y Psicología, Universidad Autónoma de Baja California, Tijuana 22390, Mexico; 4 Facultad de Ciencias del Deporte, Universidad Autónoma del Estado de Morelos, Av. Universidad No. 1001 Col. Chamilpa, Cuernavaca 62209, Mexico; 5 Facultad de Nutrición, Universidad Autónoma del Estado de Morelos, Iztaccíhuatl 100 Col. Los Volcanes, Cuernavaca 62350, Mexico; 6 Área Académica de Nutrición, Instituto de Ciencias de la Salud, Universidad Autónoma del Estado Hidalgo, Circuito Ex Hacienda La Concepción S/N, Carretera Pachuca-Actopan, San Agustín Tlaxiaca 42160, Mexico

**Keywords:** multisystem inflammatory syndrome, children, nutraceutical compounds

## Abstract

Multisystem inflammatory syndrome in children (MIS-C) has been widely reported in some children diagnosed with SARS-CoV-2. Clinical signs of MIS-C are manifested at 2 to 4 weeks after SARS-CoV-2 infection, where elevated biomarkers of inflammation and cardiac dysfunction are the hallmark of this syndrome when infection or exposure to SARS-CoV-2 has been confirmed. However, after two years of acknowledgment, MIS-C treatment is still under research to reach safety and effectiveness in the acute phase in children. Therefore, in this review, we discuss the potential use of natural compounds with antioxidant and anti-inflammatory effects to reduce collateral damage caused by hyperinflammation in MIS-C pathology for new research in treatment and interventions.

## 1. Introduction

Early reports showed that children infected with the severe acute respiratory syndrome coronavirus 2 (SARS-CoV-2) presented mild clinical symptoms or were asymptomatic [1]. Subsequently, children and adolescents who required intensive care unit admission during or after coronavirus disease (COVID-19) were described with an unusual clinical picture marked by fever and high levels of inflammatory markers [2,3,4,5]. As a result, the Royal College of Paediatrics and Child Health (RCPCH) recognized this condition as “pediatric multisystem inflammatory syndrome temporarily associated with SARS-CoV-2” (PIMS-TS), as well as “multisystem inflammatory syndrome in children” (MIS-C) identified by the U.S. Centers for Disease Control and Prevention (CDC) and the World Health Organization (WHO) [6] based on initial laboratory tests, including positivity by SARS-CoV-2 infection as a principal component. Therefore, for this review, we will refer to this syndrome as MIS-C.

First observational studies overlap MIS-C outcomes with other pediatric inflammatory diseases such as Kawasaki disease (KD), bacterial toxic shock syndromes, bacterial sepsis, and macrophage activation conditions [4,7,8]. MIS-C etiology is poorly understood, and medical care is based on KD recognition because laboratory biomarkers are comparable, and pharmacological treatment with intravenous immunoglobulins (IVIG) and aspirin has been beneficial in patients with MIS-C [9]. Nonetheless, nutritional support during and post-hospitalization has been poorly approached in this condition.

The use of nutraceuticals in several diseases has been explored due to nutrition and therapeutic potentials [10]. Nutraceuticals are generally understood as purified products derived from human food with health benefits, including the prevention and treatment of diseases [11]. Dietary supplements are ingested products with beneficial physiological effects but are not essential to the diet [12]. Alternative supportive treatments for the prevention and mitigation of COVID-19 infection and hyperinflammation have been explored, including micronutrient supplementation or nutraceutical interventions with interesting results in adults [13,14]. However, the information about its use in MIS-C is still limited. Therefore, in this review, we discuss the potential use of nutraceuticals to mitigate the inflammatory effects of MIS-C based on the acknowledgment of cellular and clinical mechanistic data for KD, COVID-19, and MIS-C to date.

## 2. MIS-C Case Definition and Clinical Manifestations

In April 2020, the Paediatric Intensive Care Society recognized a critically ill in children with characteristics of hyperinflammatory shock and evidence of SARS-CoV-2 infection. The RCPCH introduced the term PIMS-TS and subsequently, the CDC and WHO published case definitions for MIS-C in May 2020 [15,16].

**Figure 1:**
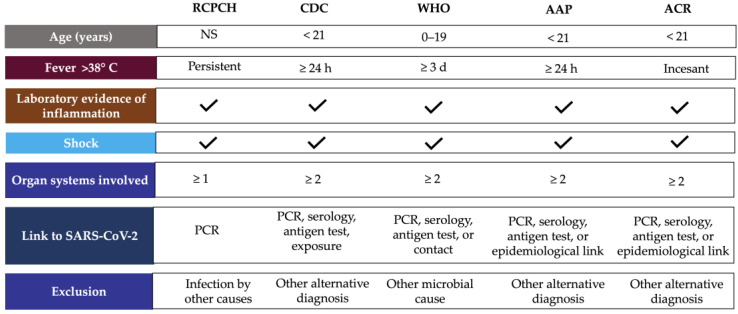
MIS-C case definition according to international guidelines. Abbreviations: AAP, American Academy of Pediatrics; ACR, American College of Rheumatology; CDC, Centers for Disease Control and Prevention; NS, no specified; PCR, polymerase chain reaction, RCPCH, Royal College of Paediatrics and Child Health; WHO, World Health Organization.

According to WHO, children and adolescents (0–19 years) showing signs of MIS-C have previous persistent fever for more than three days with any two of the following conditions: external signs of inflammation (rash or bilateral non-purulent conjunctivitis and oral cavity, hand, or foot alterations), hypotension or shock, cardiac abnormalities, signs of coagulopathy, or acute gastrointestinal conditions [17]. CDC considers MIS-C for individuals aged <21 years presenting fever for at least 24 h, laboratory evidence of inflammation, evidence of clinically severe illness requiring hospitalization, and organ involvement (respiratory, cardiac, renal, hematologic, gastrointestinal, dermatologic, or neurological) [18]. These clinical symptoms require accompaniment by laboratory findings of inflammation such as erythrocyte sedimentation rate (ESR), C-reactive protein (CRP) or procalcitonin (PCT) levels [19], and evidence of SARS-CoV-2 infection or contact with COVID-19 patients ruling out bacterial sepsis, staphylococcal or streptococcal shock syndromes caused by infection with other pathogens [3]. Only CDC considers hospitalization time as a criterion for MIS-C definition, and RCPCH did not include SARS-CoV-2 positivity or epidemiologic link (Figure 1) [16].

The American Academic of Pediatrics (AAP) defined MIS-C as the syndrome of an individual under 21 years of age presenting fever, laboratory evidence of inflammation, and proof of clinically severe disease requiring hospitalization, with multisystem (≥2) organ involvement (cardiac, renal, respiratory, hematologic, gastrointestinal, dermatologic, or neuro-logic), without a plausible alternative diagnosis, and positive for current or recent SARS-CoV-2 infection by RT-PCR, serology, or antigen testing; or exposure to COVID-19 within the four weeks before the onset of symptoms [20]. In addition, the American College of Rheumatology (ACR) published clinical guidance to define a case of MIS-C, which includes: incessant fever (greater than 38 °C), epidemiological link to SARS-CoV-2, and at least two suggestive clinical features (rash, changes in oral mucosa, conjunctivitis, neurological symptoms, edema of hands/feet) (Figure 1) [21].

Clinical signs of MIS-C appear 2–4 weeks after SARS-CoV-2 infection, with a significant proportion (75%) of antibodies to class-switched viral antigens indicating that most, if not all, cases of MIS-C are the result of previous or unclear SARS-CoV-2 infection [5,22,23]. However, the range of SARS-CoV-2 virus detected by real-time polymerase chain reaction (RT-PCR) is widely spread among children with MIS-C. It varies from 21% to 40% in studies involving either method for the detection of SARS-CoV-2 [24,25,26,27].

The diagnosis method for SARS-CoV-2 infection is also controversial. RT-PCR and antigen detection are relative indicators of viral load. SARS-CoV-2 spike (S) antigens were detectable in the blood of children with MIS-C [28]. However, N and S antigens in acute COVID-19 did not correlate strongly with RT-PCR [29]. On the other hand, the use of a novel method (MSD S-PLEX CoV-2 N and S assays) demonstrated that, during the early hospital course, SARS-CoV-2 N and S antigens are detectable in blood in most pediatric patients with acute COVID-19, but in few cases of MIS-C [30]. Therefore, the RT-PCR method for COVID-19 detection is not exclusive to MIS-C diagnosis, and serology and epidemiological linkage are also considered. Currently, the ACR emphasizes that MIS-C diagnosis should be confirmed on the basis of the totality of history, physical examination, and laboratory studies [21].

Like KD, patients with MIS-C have different features of cardiac dysfunction, such as valvulitis, coronary artery dilatation, myocardial dysfunction, and myocarditis [31,32,33]. In severe cases of MIS-C, patients require cardiac or respiratory support [34,35]. Therefore, cardiac biomarkers and echocardiography should be monitored during the hospital stay. The ACR has recommended monitoring troponin T and B-type natriuretic peptide (BNP)/N-terminal proBNP (NT-proBNP) and assessment of BNP/NT-proBNP levels to distinguish between MIS-C patients with and without left ventricular (LV) dysfunction [21]. However, a meta-analysis of laboratory cardiac markers for children with MIS-C and COVID-19 revealed that only BNP was the key cardiac marker that showed differences between patients with non-severe MIS-C and severe COVID-19 and between non-severe and severe MIS-C patients. Meanwhile, neither troponin nor aspartate aminotransferase showed notable differences in cardiac injury between MIS-C and COVID-19 patients [36]. Nevertheless, coronary artery aneurysms regressed in the first month in 80% of patients with MIS-C, and this was not observed in KD patients [21,24,37].

Furthermore, MIS-C differs from KD concerning the age at presentation, as MIS-C typically affects the oldest children and adolescents (with a range of 6 to 12 years), unlike KD, which is more common before the age of 5 years [3,27,37,38]. Other interesting findings include that severe manifestations of MIS-C occur less frequently in Caucasians compared to the frequency expected in the general population (many of whom are of African-American or Afro-Caribbean ethnicity) [23,38,39]. In addition, the ACR panel considers that patients with MIS-C more commonly manifested LV dysfunction, shock, gastrointestinal, and neurological symptoms than patients with KD [21].

## 3. Inflammatory Markers in MIS-C

Even though the immunopathologic mechanisms of MIS-C remain poorly understood, high inflammatory markers have been identified, and patients with MIS-C were found to respond appropriately to therapy with immunomodulators or anti-inflammatory drugs [40,41,42,43]. Due to the clinical course of MIS-C and its high variability, identification of the distinct cellular, chemokines, cytokines, coagulation, and inflammatory markers is essential to comprehend clinical evolution. In addition, it has been suggested that cells involved in the innate and adaptive immune response are affected, as well as important markers of coagulation and cardiac and hepatic function [44].

Most children with MIS-C presented anti-SARS-CoV-2 IgG antibodies, indicating a past infection of at least 2–3 weeks (Table 1) [40]. The study by Anderson et al. [45] also suggests that children with MIS-C have high SARS-CoV-2 spike immunoglobulin G (IgG) titers compared with children with severe COVID-19. In addition, autoantibodies directed against endothelial, gastrointestinal, and immune cells were found [46].

The first class of clinical parameters reported associated hyperinflammation, including elevated acute phase reactants [5,41,44,47], accompanied by increased biomarkers of coagulation [41,47,48,49] and cardiac function [39,43,50,51]. In the acute phase of MIS-C, exacerbation of cytokines as some interleukins (IL), tumor necrosis factor-alpha (TNF-a), and interferon-gamma (INF-γ) levels have been reported [48,51,52,53,54,55], as well as chemokines including the IL-2 receptor agonist, C-C motif chemokine ligand 2 (CCL2), C-X-C motif chemokine ligands 8, 9 and 10 (CXCL8, CXCL9, CXCL10), and monocyte chemoattractant protein (MCP)-1 [42,48,56,57,58]. In addition, changes in leukocyte count and distribution are considered as circulating biomarkers [54,59,60,61,62], as well as significant changes in serum biomarkers such as albumin [26,44,63,64], lactate dehydrogenase (LDH) [41], creatinine [41,48], sodium [48,57,63], triglycerides [65,66] and zonulin [67,68] (Table 1).

**Table 1:** MIS-C circulating biomarkers altered.

Category	Biomarkers	References
Antibodies	Anti-spike IgG e IgA	[46]
Acute phase reactants	↑ C-reactive protein, procalcitonin, ferritin, erythrocyte sedimentation rate	[5,41,44,47]
Coagulation	↑ D-dimer, fibrinogen, prothrombin T, partial thromboplastin time	[41,47,48,49]
Cardiac function	↑ Troponin, brain type natriuretic peptide (BNP), Pro-BNP	[39,43,50,51]
Cytokines	↑ IL-1a, IL-2, IL-6, IL-8, IL-17, IL-33, TNF-a, IFNγ	[48,51,52,53,54,55]
Chemokines	↑ CCL2, CXCL8, CXCL9, CXCL10, MCP-1	[42,48,56,57,58]
Monocytes	↓ Monocyte HLA-DR and CD86+	[52,69]
Dendritic cells	↓ Plasmacytoid dendritic cells	[56,69]
Platelets	↓ Total count of platelets	[24,50,53,70]
Neutrophils	↑ Total count of neutrophils	[24,59,60,61,62,71]
Natural killer	↓ CD16+, CD56+ ↑ CD38+	[60,69,72]
Lymphocytes B	↑ Plasmablasts, naive B cells	[59,60,73]
Lymphocytes T	↓ CD4+, CD8+	[52,62,73,74,75]
Other laboratory markers	↓ Albumin, sodium ↑ Lactate dehydrogenase, alanine transaminase, creatinine, triglycerides, creatine kinase, blood urea nitrogen, zonulin	[26,41,44,45,48,63,64,65,66,67]

Upward arrows indicate increased biomarker levels. Down arrows indicate decreased biomarker levels.

MIS-C and severe COVID-19 have prominent systemic inflammation [68]. However, recent evidence suggests some differences in the inflammatory profile in MIS-C versus COVID-19, as analyzed by Zhao et al. [54], where children with MIS-C had lower levels of LDH, total platelet count (PLT) and higher levels of ESR compared to children with severe COVID-19. In contrast, lower levels of absolute lymphocyte count (ALC) and higher levels of CRP, D-dimer, and absolute neutrophil count (ANC) were observed in patients with MIS-C compared to non-severe COVID-19. In addition, patients with severe MIS-C had increased levels of leukocytes, CRP, D-dimer, and ferritin compared to non-severe MIS-C. In addition, MIS-C showed higher levels of CRP, D-dimer, ferritin, and creatinine and low levels of leukocytes, ALC, PLT, albumin, and sodium versus KD [64]. Therefore, the evolution of inflammatory markers could be useful in order to assess the severity of MIS-C [72].

Further research on principal pathways to induce cytokine storm revealed that during the acute phase of MIS-C, altered antigenic presentation, measured by major histocompatibility complex (MHC) II cell surface receptor (HLA-DR) and CD86 expression, was observed, although γδ T cells and CD4+CCR7+ T cells were activated [52,62]. Activation markers of 4+ and CD8+ TCD cells were positively correlated with disease severity [75,76]. A consistently high frequency of these markers was identified in MIS-C, suggesting T-cell activation and proliferation, particularly of CD8+ T cells with the expansion of T-cell receptor Vβ 21.3-expressing cells, which is a suggested signature of MIS-C, because this was not observed in KD, TSS or acute COVID-19 [60,73,77]. Therefore, the authors suggest that the innate and activating T-cell response could be dominant in the acute phase. Still, during the resolution of MIS-C, there is a predominant effect of regulatory T cells, and this could be taken into account for the immunomodulatory treatment depending on the disease course [52].

Ramaswamy et al. [59] profiled MIS-C, adult COVID-19, and healthy pediatric and adult individuals to identify a signature in MIS-C patients. The authors found high expression of alarmin-related S100A genes in monocytes and neutrophils that could be mediating part of the inflammatory response observed in MIS-C. In addition, they found higher expression of perforin, granzyme A, and H in natural killer (NK) cells in MIS-C versus healthy pediatric donors. Reduced expression of cytotoxic molecules such as granzyme B [78,79] and perforins [62] have been described in the adult population with COVID-19, but in children with MIS-C, both NK cells and CD8+ T cells exhibited elevated cytotoxicity with potential relevance to tissue damage through pyroptosis pathways [63].

Hoang and colleagues [65] reported low CD16+CD56+ expression in children with MIS-C compared to children with COVID-19, suggesting NK cell cytopenia. Moreover, Vella et al. found that 80% of NK cells presented CD38+, suggesting an activation of the innate response in addition to the adaptive immune defense mediated by CD4+ and CD8+ T cells [60]. These data indicate that, even though there is a decrease in the number of NK cells, there is an increase in its activity and functionality.

Regarding B cells, effector B cells, and class-switched memory B cells, a decrease in blood and an increase in circulating plasmablasts have been observed, which could have a potential humoral response suggesting a potential target for MIS-C recovery [37,40,52,59,73,80,81].

Recent research employing proteomics revealed changes in complement activation and coagulation pathways in the plasma of MIS-C and COVID-19 with acute respiratory distress syndrome (ARDS). The MIS-C phenotype activated the Fc receptor γ (FcGR) and B-cell receptor (BCR) pathways. FcGR receptors are crucial for an antibody-mediated immune response, suggesting a solid implication of antibodies in the progression of MIS-C [68]. Furthermore, Fc receptors may indicate the IVIG treatment received by patients with MIS-C, as previously established [82].

## 4. MIS-C Treatment

MIS-C treatment focuses on the clinical stabilization of hospitalized patients and the prevention of multi-organic damage and long-term sequelae; for non-hospitalized patients, antiplatelet agents have shown promising results. The ARC recommends using aspirin for 3–5 mg/kg/day in patients without bleeding [21].

MIS-C treatment in hospitalized patients is based on the KD approach, focused on the use of IVIG and glucocorticoids [83,84,85,86,87]. The AAP, ACR, American Heart Association (AHA), Helen DeVos Children’s Hospital Foundation (HDVCH), and Infection Diseases Society of America (IDSA), as well as other worldwide health organizations, recommend the continued use of IVIG at a dose of 1–2 g/kg, steroid therapy (2–3 mg/kg/d), and antiplatelet therapy (aspirin) [88].

The use of IVIG has been recommended in KD patients to reduce coronary artery abnormalities [89,90], while its benefit in myocarditis remains unclear because the successful use of IVIG in coronavirus-associated myocarditis has been supported only by case reports [21]. The American Heart Association (AHA) suggests that, although the mechanism of action of IVIG is unknown, there is a modulation of cytokine production, neutralization of toxins, augmentation of regulatory T-cell activity, and regulation of antibody synthesis [91,92].

**Figure 2:**
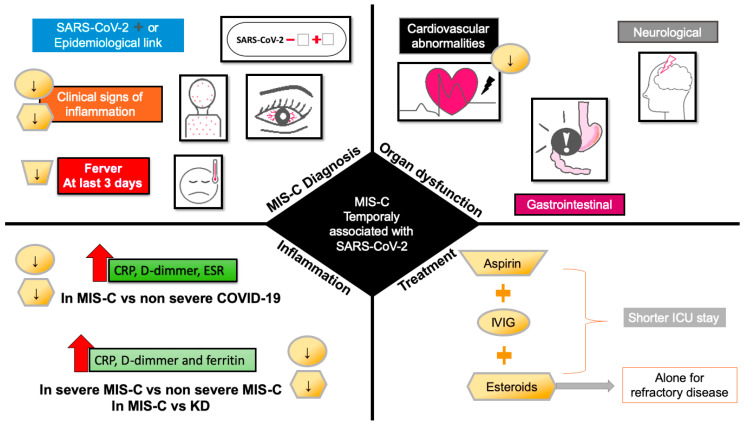
Effect of pharmacological treatment for MIS-C on clinical signs at admission, organ dysfunction and inflammation. Downward arrows indicate the decrease in signs, symptoms, and markers of inflammation by treatment with aspirin (trapezoid), IVIG (circle), and steroids (hexagon). Abbreviations: CRP, C-reactive protein; ESR, erythrocyte sedimentation rate; ICU, intensive care unit; IVIG, intravenous immunoglobulin.

Using glucocorticoids in combination with IVIG is more effective than monotherapy with IVIG in patients without contraindications to glucocorticoids and is associated with shorter ICU stays [50,93]. The monotherapy with glucocorticoids needs more investigation, and the experts do not recommend its use alone until more evidence is feasible (Figure 2).

The use of antibiotic therapy is only recommended by AAP for mild and severe illness or shock. Anticoagulant therapy is suggested as prophylaxis or therapy in patients with eject fraction <35% or thrombosis evidence [88]. In patients with a higher risk of complications by IVIG or refractory disease, the experts recommend the intensification with higher doses of glucocorticoids, as well as the use of anakinra, a recombinant human IL-1 receptor antagonist [91,92,94,95], or the TNF-a inhibitor infliximab [21,93,96].

## 5. Nutraceuticals, Alternative or Complementary Therapy?

In order to discuss the use of nutraceuticals in a disease context, it is imperative to describe its definition and different conceptualization versus functional food and dietary supplements. In this sense, Stephen DeFelice coined the term nutrition and pharmaceutical as a nutraceutical in 1986. In 2003, Karla referred to functional food as “food that is being cooked or prepared using scientific intelligence, with or without knowledge of how or why it is being used. If this preparation is used for prevention or treatment of disease, it is called nutraceutical” [97]. The Oxford English Dictionary defines functional food as a foodstuff containing chemical or biological additives to produce a beneficial physiological effect on the consumer, as well as nutraceutical [12]. While the term “dietary supplement” refers to a product added to a diet that bears vitamins, minerals, amino acids, or any other ingredient that supplements the diet by increasing the total daily intake [97].

Despite the nutraceutical term has been applied indistinctly to functional food and dietary supplement, the discussion about it reveals the need for an integrative definition based on scientific evidence as well as was discussed by Aronson in 2017 [12], based on the DeFelice lecture in 2014 about the term nutraceutical and the scientific evidence, highlighting that study design and cell demand may influence negative results. According to the interest of this review, we will discuss the use of nutraceuticals as purified products derived from human food with health benefits, including the prevention and treatment of disease, which includes the use of dietary supplements and functional foods.

Nutrition interventions in health and disease have been widely implemented in community settings using complementary and alternative medicines. In the European Union, the use of non-pharmacological interventions has been raised in the last decades. These strategies are often applied to offset the use of conventional drugs [98]. However, its uses as a complementary or alternative therapy are still controversial and influenced by experiences and perceptions more than the knowledge of scientific evidence.

In a recent systematic review of the potential factors that influence the use of complementary and alternative medicine, the top reasons were having good expectations of its benefits and safety and dissatisfaction with conventional medicine. In addition, illnesses such as cancer, diabetes, cardiovascular disease, and human immunodeficiency virus were associated with the acceptability of the use of complementary medicine, where commonly used drugs cannot be satisfactorily effective, and people tend to seek this therapy as a way of meeting their needs or filling a gap left by conventional medicine [99].

Likewise, in the study of the factors that affect consumers’ decision to take nutraceuticals, the experience of those who have used these products may have contributed to the positive perception of its health benefits. In addition, people who perceived that nutrient intake was inadequate from diet alone decided to take nutraceuticals. On the other hand, the high cost of nutraceuticals and their lack of knowledge were barriers to their use. An interesting aspect is that consumers rarely mention clinical evidence because of a lack of access to clinical evidence due to obstacles to understanding scientific literature. Therefore, the primary resource of information were friends, family, and mass media, more than healthcare professionals [100].

In other public surveys, people were more inclined to use nutraceuticals or dietary supplements if a registered medical practitioner prescribed them. Still, clinicians have divided opinions because most consider nutraceuticals relatively safe for consumption, but they recognize the need to undergo documented clinical trials such as pharmaceuticals [101]. However, many health professionals recommend using nutraceuticals influenced by their personal use or work experience and not by high-quality information sources [102]. The use of nutraceuticals for health and disease remains controversial and limited by the gap between clinical evidence and users’ knowledge. Consequently, nutraceutical researchers need to increase efforts in the critical choice of the compounds to be tested, the study design, the scope of the results, and its proper and simplified communication to users.

## 6. Nutraceuticals in Inflammatory Diseases and COVID-19

**Figure 3:**
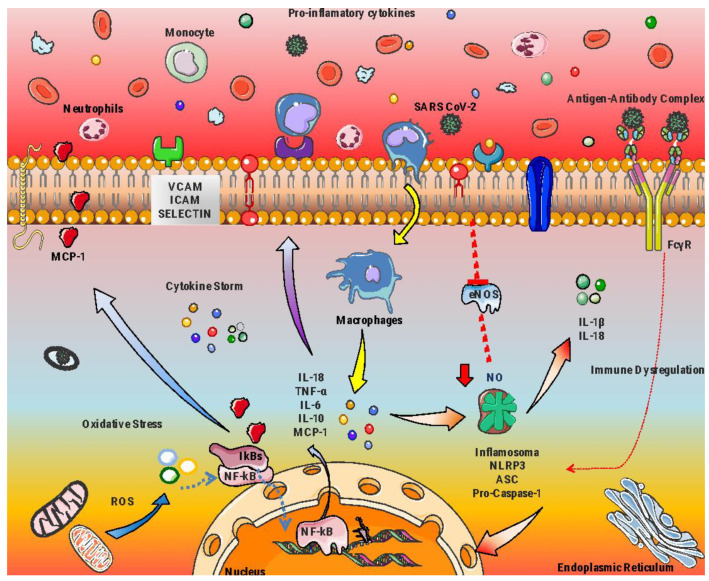
Hyperinflammatory state and oxidative stress in immunological response in MIS-C after SARS-CoV-2 infection.

The etiology of different chronic and degenerative diseases is widely reported to be related to persistent and dysregulated inflammation [103,104,105]. Indeed, this process emerges as the key player at molecular and cellular levels for the appearance and development of subsequent chronic inflammation-related disorders, such as cardiovascular diseases, hypertension, diabetes, and pulmonary diseases, including COVID-19 and MIS-C (Figure 3) [106,107,108,109].

Recently, interest in functional foods for treating these conditions has increased, mainly antioxidant-rich foods and other bioactive compounds focused on potential pharmacological activities. Modern medicine develops new drugs and therapeutic supplements against chronic and viral diseases and avoids severe inflammation [110,111,112]. It is well known that a healthy diet can enhance the immune system and reduce injury induced by chronic or acute inflammatory processes. In addition, the deficit in the consumption of exogenous antioxidants and the decrease in the endogenous antioxidant system has been linked to alterations in body functions [113]. In this context, studies have shown that several foods and natural products are important sources of bioactive compounds such as nutraceuticals, vitamins, and micronutrients with anti-inflammatory [114], antioxidant [115], antithrombotic [116], antidiabetic [117] and antidiuretic [118] activities that play an important role in health care. Therefore, in this review, we have suggested that the intervention with functional foods could be a potential therapeutic tool to reduce SARS-CoV-2-induced inflammatory responses and the long-term effects it would have produced, as MIS-C [119,120].

Given that hyperinflammation in MIS-C is associated with SARS-CoV-2 infection, it is important to know non-pharmacological interventions in clinical trials to reduce inflammation during COVID-19 or improve patient prognosis during hospitalization. In the search for nutraceutical compounds as adjuvants in KD, TSS, or COVID-19 in children, nutraceutical interventions have only been carried out in adult subjects with COVID-19. We found only one intervention with vitamin C in children with KD to evaluate changes in the diameter of the brachial artery [121]. In clinical trials, the most commonly used nutraceuticals with anti-inflammatory and antioxidant properties tested as complementary treatment versus COVID-19 were curcumin, omega-3 fatty acids, quercetin, and vitamins A, C, and D3 (Table 2).

## 7. Potential Nutraceutical Compounds for MIS-C

**Figure 4:**
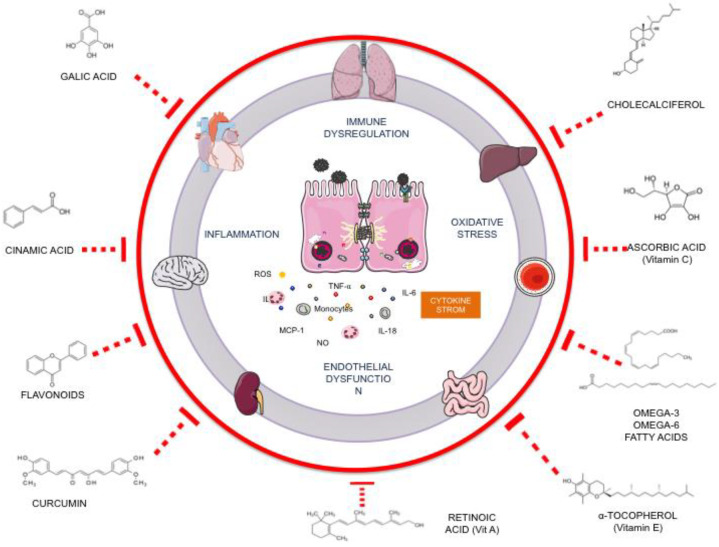
Potential nutraceutical compounds for complementary treatment of MIS-C by its antioxidant and anti-inflammatory properties to regulate cytokine storm and reduce organ damage.

MIS-C is characterized by a hyperinflammatory state featuring loss of tissue homeostasis and endothelial dysfunction due to cytokine storm that develops an oxidative stress environment and promotes tissue damage leading to multisystem failure. Based on nutraceuticals used for COVID-19 treatment, we discuss its potential action as a complementary treatment for hyperinflammation in MIS-C (Figure 4).

### 7.1. Curcumin

Currently, one of the most studied phytochemicals in the field of anti-inflammatory diseases is curcumin (1,7-bis(4-hydroxy-3-methoxyphenyl)-1, 6-heptadiene-3, 5-dione) [149], a non-toxic natural polyphenol that also exhibits antioxidant properties. A wide range of studies highlights its beneficial effects on cardiovascular disease [150], diabetes [151], rheumatoid arthritis [152], and inflammatory bowel diseases [153]. Many studies have shown that curcumin can regulate transcription factor kappa B (NF-κB), mitogen-activated protein kinase (MAPK), transcription factor-activated protein 1 (AP-1), and protein kinase serine/threonine (AKT) signaling pathways [154,155,156]. Curcumin also suppresses or downregulates the expression of some growth factors and cytokines such as TNF-α, IL-1, IL-6 and IL-8, epidermal growth factor, estrogen receptors, and adhesion molecules (VCAM-1 and ICAM-1) [157,158,159] and plays an important role in pulmonary edema induced in COVID-19-related lung conditions [160]. Moreover, in the natural course of COVID-19, pneumonia-like symptoms appear, which increase acute lung injury due to respiratory stress, which is reduced by curcumin supplementation [161,162]. However, one limitation of its application in healthcare is that curcumin has low solubility and poor bioavailability, which has been demonstrated in rats, mice, and human studies [163,164]. The use of nanoparticles in clinical trials has been a promising therapy for increasing bioavailability and modulation of transcription factors that regulate inflammation [122].

### 7.2. Omega-3 Fatty Acids

On the other hand, several studies have shown that consumption of omega-3 fatty acids, such as α- linolenic acid (ALA), eicosapentaenoic acid (EPA), and docosahexaenoic acid (DHA), can reduce inflammation processes [165,166,167,168,169,170]. In addition, high consumption of omega-3 PUFAs results in the reduction of proinflammatory cytokines such as IL-1β, IL-6, IL-8, and TNF-β; inhibits adhesion molecules expression (VCAM, ICAM, and selectins) [171] and other important anti-inflammatory mechanisms including regulating cell membrane phospholipids composition [172], modulation of lipid rafts implicated in viral infections [173] decreasing expression of proinflammatory genes by inactivation of NF-κB [174] and acting like a metabolic regulator of inflammatory responses through G-protein-coupled receptor 120 (GPR120) [175]. In addition, it has demonstrated that the production of DHA derivatives, known as immunological mediators such as resolvins, maresins, prostaglandins, thromboxanes, leukotrienes, and protectins [173,176], could be a novel way to regulate inflammation through the expression of IL-1β and TNF-α and limiting tissue infiltration by neutrophils [177,178]. Moreover, omega-3 main antiviral properties are related to the cell lipid metabolism in the replication cycle and modulate interferon activity [179]. In adult patients with COVID-19, the use of EPA and DHA reduces CRP and ESR levels and body pain when administered with hydroxychloroquine [125]. In addition, when used alone, it could increase the survival rate [126].

### 7.3. Vitamins

Several studies have demonstrated an inverse association between vitamin intakes, such as A, C, D, and E, and the risk of inflammatory disorders related to cardiovascular and respiratory diseases, including COVID-19. This could be due to its antioxidant capacity for scavenging the oxidative free radicals and its anti-inflammatory properties contributing to restoring endothelial function. Vitamin A (in its multiple forms; retinoic acid, retinol, retinal) is an essential micronutrient that increases angiogenesis. Reparative collagen synthesis also plays an important role in intestinal immunity and epithelial integrity and promotes healthy colonization of the intestinal mucosa with commensal bacteria [180,181,182]. These retinoids bind to specific receptors in the cytoplasm and the nucleus, thus affecting cell division, differentiation, RNA and protein synthesis, and lysosome-membrane stabilization [183]. These actions are primarily mediated by all-trans-retinoid acid, 9-cis retinoic acid, and 13-cis retinoic acid. All-trans retinoic acid is a represent ligand for the family of nuclear retinoic acid receptors (RARα, β, and γ) and retinoid X receptors (RXRα, β, and γ), the latter being also activated by 9-cis retinoic acid and 13-cis retinoic acid [184].

The deficiency of vitamin A and its metabolites is a public health problem that can cause micronutrient malnutrition, disturb the microbiota symbiosis, slow growth, impair innate immunity, and have adverse health consequences for people and animals [185,186]. On the other hand, a reduction in circulating vitamin A concentration leads to an elevated release of proinflammatory cytokines such as IL-6, IL-1β, and TNF-α. At the hepatic level, this excess of cytokines increases CRP and alpha-1-acid glycoprotein (AGP) [187]. On the other hand, in COVID-19 patients, vitamin A plasma levels are reduced, which is related to acute respiratory syndrome due to vitamin A having immune regulatory functions [188]. It also reduces fever, body aches, weakness and fatigue, paraclinical symptoms, WBC count, and CRP levels [130]. However, excessive utilization of vitamin A often results in local and systemic toxicity [189].

On the other hand, vitamin C or ascorbic acid protects against atherogenesis by inhibiting LDL oxidation, impairing the production of ROS by vascular cells, and limiting cellular responses to oxidized LDL. In addition, this acts as a cofactor for prolyl hydroxylase in the extracellular matrix, the enzyme responsible for collagen biosynthesis, and it has also been considered an anti-cancer agent. In addition, vitamin C has an immune modulator function, which could be explained by the present high intracellular concentrations in lymphocytes [190]. Two action mechanisms of ascorbic acid have been described. The ascorbic acid (shape reduced) enters cells using sodium-dependent vitamin C transporters. In contrast, the dehydroascorbic acid (oxidized form) enters cells via glucose transporters (GLUTs) and then interacts with different enzymatic systems involved in the regulation of numerous biological processes [190,191,192].

Humans cannot synthesize ascorbic acid; therefore, excellent sources of vitamin C are citrus fruits, berries, tomatoes, potatoes, and green leafy vegetables. Nevertheless, even though vitamin C is reported to present anti-inflammatory properties increasing the secretion of proinflammatory cytokines such as IL-10, this is exacerbated by accumulation in leukocytes, decreasing the levels of cytokines such as interferon-y, TNF-a, and IL-6, especially in patients with pneumonia. In vitro studies also report that vitamin C has proinflammatory properties, promoting the proliferation of fibroblasts and lymphocytes, phagocytosis processes, and the generation of ROS [193,194]. This vitamin deficiency has been associated with impaired immunity. In addition, under conditions of physiological stress, including infection, trauma, and surgery, reduced plasma levels and administration reduces monocyte adhesion. However, vitamin C consumption increases interferon production and upregulates immunological cells (lymphocytes and natural killer cells) as an antiviral response [195,196]. In children with KD, the intravenous infusion of vitamin C increased the percent change in the brachial artery diameter induced by reactive hyperemia [121]. Therefore, it could be considered for cardiovascular improvement in MIS-C, but clinical evidence is needed.

Other vitamins with cardio and respiratory protection characteristics are vitamin E or α-tocopherol. The supplementation with vitamin E inhibits proinflammatory cytokine production, modulates adhesion molecule expression and endothelium-derived NO synthesis, and improves innate natural killer (NK cell) response [195]. Moreover, vitamin E intake gives protection of cell membrane integrity from the detrimental effects of free radicals [197]. The use of vitamin E combined with vitamins A, C, B, and D, reduced ESR, CRP, IL-6, TNF-a, and hospitalization time in adults with COVID-19 [131].

The possible role of vitamin D in SARS-CoV-2 infection in children could be explained initially by its antiviral activities and its use in adults with COVID-19 [134,135,136,137,138,139,140,141,142,143,144,145,146,147,148]. It has been postulated that vitamin D intake reduces inflammatory state and plays an essential role in endothelial function, mediated by vitamin D receptors (VDRs). Many studies have shown an association between vitamin D and CRP, IL-6, and IL-10 ratio [198]. Vitamin D deficiency has been reported in several chronic conditions associated with increased inflammation and dysregulation of the immune system [199,200,201]. Vitamin D modulates immune function too. Vitamin D receptor (VDR) is expressed by most immune cells, including B and T lymphocytes, monocytes, macrophages, and dendritic cells. The signaling of vitamin D and VDR together has an anti-inflammatory effect [202]. Some studies have reported that vitamin D treatment could be helpful for COVID-19 prevention [140,146] or treatment because vitamin D plays an essential role as a modulator of immunocompetence [203,204,205], regulates B and T cells [141,143], reduces CRP levels [141], mortality [135,136,139], the time of hospital stay [142,143] and the need of oxygen support [142,148]. However, there is no consensus about doses and therapeutic schemes because of high variability among clinical trials. The classic functions of vitamin D are to regulate calcium-phosphorus homeostasis and to control bone metabolism. Still, recent studies showed that severe vitamin D deficiency in children with MIS-C increased the risk of cardiovascular events [206].

### 7.4. Polyphenols from Pomegranate

Another rich source of a wide variety of bioactive compounds with anti-inflammatory properties is pomegranate *(Punica granatum),* an ancient fruit used in traditional medicine in several cultures and also gained considerable recognition as a functional food in the modern era. Several studies suggested that pomegranate can exert antiatherogenic [207], antidiabetic [208], antioxidant [209], antihypertensive [210], anti-inflammatory effects, and regulate lipid metabolism in metabolic-disorder-associated diseases [119]. In the last decades, different classes of phytochemicals identified from pomegranates such as ellagitannins (castalagin), flavonoids (procyanidins), lignans (punicatannin C), triterpenoids (ursolic acid), fatty acids (punicic acid), and organic acids (citric acid) has linked to health-promoting activities [211,212,213]. Most studies regarding pomegranate fruit, as well as its different compounds (peel powder, juice, extract, and oil), exert health benefits in respiratory conditions such as lung cancer, asthma, chronic obstructive pulmonary disease, and alveolar inflammation inhibiting the production and downregulation of the expression of proinflammatory cytokines and modulates NF-κB, Nrf2, NLRP3 and MAPK pathways [214,215,216,217,218,219]. These beneficial effects are attributed to its constituents, ellagic acid, ellagitannins anthocyanins, and ellagic acid acting individually or synergistically. In addition, pomegranate juice has a potent antiviral activity, which has been proven in HIV and various influenza types research, linking the role of glycoproteins and pomegranate chemical compounds; additionally, fresh pomegranate juices inhibit the replication from these viruses [220,221].

## 8. Conclusions

After more than two years of the COVID-19 pandemic, the pathomechanisms of MIS-C are not yet fully understood, and it is well-recognized that cytokine storm is a key to organ dysfunction. Therefore, the use of IVIG and steroids are the pharmacological recommended treatments. However, complementary therapy based on natural compounds could be feasible by its potential antioxidant and anti-inflammatory activities. As many combinations could have these actions, the perspective of the use of nutraceuticals used for COVID-19 in adults as curcumin, omega-3 fatty acids, and vitamins (A, C, D, and E), have been shown promised results by their ability to reduce inflammatory markers and better prognostic during the hospital stay. Therefore, future clinical trials are needed to support MIS-C treatment with natural compounds.

## Figures and Tables

**Table 2:** Clinical trials evaluating the effects of nutraceuticals in COVID-19 and KD.

Compound	Population/ Disease	Treatment	Study Design	Results Intervention vs. Control	Ref.
Curcumin	40 adults COVID-19	I = nano-curcumin capsules—160 mg/day/2 weeks	Triple-blind, placebo-controlled, RCT	↓ Transcription factor that controls Th1 cytokine and INF-g on day 7	[122]
	48 adults COVID-19	I = nano-curcumin capsules—160 mg/day/6 days	Double-blind, placebo-controlled, RCT	↓ Milder symptoms	[123]
	46 outpatients (adults) COVID-19	I = capsules with 500 mg of curcumin + 5 mg piperine	Double-blind, placebo-controlled, RCT	↓ Weakness	[124]
Omega-3 fatty acids	Adults with COVID-19	I = hydroxychloroquine + 2 g of DHA + EPA for 2 weeks C = hydroxychloroquine	Single-blind, controlled, RCT	↓ Body pain, fatigue, appetite, ESR, CRP	[125]
	128 adults Severe COVID-19	I = one capsule of 1000 mg/14 days	Double-blind, RCT	↑ Survival rate and higher levels of arterial pH, HCO_3_ ↓ BUN, Cr, and K	[126]
Palmitoylethanolamine (PEA)	Unvaccinated adults recently infected with COVID-19	I = 1.2 g of PEA daily C = placebo tablets daily/4 week	Double-blind, RCT	↓ P-selectin, IL-1β, and IL-2 concentrations	[127]
Quercetin	152 COVID-19 outpatients	I = 400 mg/daily/30 days C = without quercetin	Controlled, open- label, RCT	↓ Frequency and length of hospitalization, need for non-invasive oxygen, progression to ICU, and deaths	[128]
	42 COVID-19 outpatients	I = first 7 days with 600 mg/daily, following 7 days with 400 mg/daily C = standard care	Controlled, open- label, RCT	↓ LDH, ferritin, CRP, and D-dimer	[129]
Vitamin A	I = 91 adults Ctrl = 91 adults Infected with COVID-19	I = 25,000 IU/d oral vitamin A/10 days C = hydroxychloroquine	Triple-blind controlled trial	↓ Fever, body ache, weakness and fatigue, paraclinical symptoms, WBC count, and CRP	[130]
Vitamin A, B, C, D and E	I = 30 adults Ctrl = 30 adults COVID-19	25,000 IU daily of vitamins A, 600,000 IU once during the study of D, 300 IU twice daily of E, 500 mg four times daily of C, and one amp daily of B complex for 7 days	Single-blinded, RCT	↓ ESR, CRP, IL-6, TNF-a, and hospitalization time	[131]
Vitamin C	I = 39 children with acute KD Ctrl = 17 healthy children	I = intravenous infusion of 100 mL of 0.9% saline containing 3 g of vitamin C over 10 min C = placebo (100 mL 0.9% saline)	Placebo-controlled, RCT	↑ Percent change in diameter of the brachial artery induced by reactive hyperemia in 19 patients with a history of KD	[121]
	I = 31 adults Ctr = 31 adults COVID-19	I = 500 mg of vitamin C daily/14 days	RCT	↑ Mean survival duration	[132]
	30 adults with severe COVID-19 infection	I = single oral dose of 500,000 IU C = placebo	Open-label, RCT	No effects	[133]
Vitamin D3	218 adults mild-to-moderate COVID-19	I = single oral dose of 500,000 IU Ctrl = placebo	Multicenter, double-blind, sequential, placebo-controlled, RCT.	No effects	[134]
	207 patients ≥65 years COVID-19	I = single oral dose 400,000 IU C = standard-dose 50,000 IU	Multicenter, open-label, RCT	↓ Overall mortality at day 14. The effect was no longer observed after 28 days	[135]
	151 adults with COVID-19 and vitamin D deficiency (serum < 25 nmol/L)	I = high-dose booster (≥280,000 IU) up to 7 weeks	Retrospective	↓ Risk of COVID-19 mortality	[136]
	200 adults With moderate to severe COVID-19	I = single oral dose 200,000 IU Ctrl = Placebo	Post hoc analysis of multicenter, double-blind, placebo-controlled, RCT	No effect in cytokines, chemokines, and growth factor in hospitalized patients with moderate to severe COVID-19	[137]
	240 adults mild-to-moderate COVID-1	I = single oral dose of 200,000 IU C = placebo	Multicenter, double-blind, placebo-controlled, RCT	No effects	[138]
	95 adults COVID-19	I = 50,000 IU per month, or 80,000 IU or 100,000 IU or 200,000 IU/2–3 months, *n* = 66), or daily supplementation with 800 IU (*n* = 1). C= without vitamin D supplements (*n* = 28)	Intervention study	↑ 3-month survival in older COVID-19 patients	[139]
	129 workers COVID-19	I = 50,000 IU/week for 2 weeks, followed by 5000 IU/day for the rest of the study C = 2000/day	Intervention study	Asymptomatic SARS-CoV-2	[140]
	129 adults COVID-19	I = 100,000 IU (50,000 IU at first day and eight days of hospitalization) C = without vitamin D3	Randomized, open-label, single-center study	↓ Time of hospitalization, CRP (at day 9) frequencies of CD38++CD27 transitional and CD27-CD38+ mature naive B cells ↑ Neutrophil and lymphocyte count and CD27-CD38-levels in DN B cells	[141]
	50 adults COVID-19	I = 25,000 IU/daily/4 days, followed by 25,000 IU/week/6 weeks C = placebo	Double-blind, placebo-controlled, RCT	↓ Hospital stay and need for supplemental oxygen	[142]
	86 adults COVID-19	I = 10,000 IU/day/14 days C = 2000 IU/day/14 days	Multicenter, single-blind, prospective, RCT	↑ Anti-inflammatory cytokine IL-10, levels of CD4+ T cells ↓ Hospital stays	[143]
	106 adults COVID-19 and circulating 25(OH)D3 concentration of <30 ng/mL	I = 25 μg daily (3000 to 6000 IU per day) up to 30 and 60 days C = placebo	Multicenter, double-blind, placebo-controlled, RCT.	Correct vitamin D deficiency/insufficiency in patients with COVID-19 ↑ Blood lymphocyte percentage	[144]
	69 adults Mild to moderated COVID-19	I = 5000 IU/day/14 days C = 1000 IU/day/14 days	Multicenter, RTC	↓ Time to recovery for cough and gustatory sensory loss among patients with suboptimal vitamin D status	[145]
	321 recruited subjects for preventive treatment of COVID-19	I = 4000 IU VD/daily/30 d C = placebo/daily/30 d	Double-blind, parallel, RTC	↓ The risk of acquiring SARS-CoV-2 ↑ Serum levels of 25-hydroxyvitamin D3, independently of vitamin D deficiency	[146]
	30 old patients’ recovery after COVID-19 infection	I = 2000 IU/day/for 6 weeks C = placebo	Pilot study, double-blind trial	↑ Serum creatine kinase levels returned to optimal values	[147]
Vitamin D3 magnesium and vitamin B12	73 adults with COVID-19 without oxygen support	I = 1000 IU/d + magnesium 150 mg/d + vitamin B12,500 mcg/d	Cohort study	↓ The proportion of patients with clinical deterioration requiring oxygen support, intensive care support, or both	[148]

Up arrows indicate an increase, or down arrows a decrease, in the specified characteristics of the intervention group when compared to the control group. Abbreviations: BUN = blood urea nitrogen; C = control or compared group; I = intervention; RTC, randomized controlled trial.

## Data Availability

Not applicable.

## References

[1] Castagnoli R., Votto M., Licari A., Brambilla I., Bruno R., Perlini S., Rovida F., Baldanti F., Marseglia G.L. (2020). Severe Acute Respiratory Syndrome Coronavirus 2 (SARS-CoV-2) Infection in Children and Adolescents: A Systematic Review. JAMA Pediatr..

[2] Pouletty M., Borocco C., Ouldali N., Caseris M., Basmaci R., Lachaume N., Bensaid P., Pichard S., Kouider H., Morelle G. (2020). Paediatric multisystem inflammatory syndrome temporally associated with SARS-CoV-2 mimicking Kawasaki disease (Kawa-COVID-19): A multicentre cohort. Ann. Rheum. Dis..

[3] Ramcharan T., Nolan O., Lai C.Y., Prabhu N., Krishnamurthy R., Richter A.G., Jyothish D., Kanthimathinathan H.K., Welch S.B., Hackett S. (2020). Paediatric Inflammatory Multisystem Syndrome: Temporally Associated with SARS-CoV-2 (PIMS-TS): Cardiac Features, Management and Short-Term Outcomes at a UK Tertiary Paediatric Hospital. Pediatr. Cardiol..

[4] Toubiana J., Poirault C., Corsia A., Bajolle F., Fourgeaud J., Angoulvant F., Debray A., Basmaci R., Salvador E., Biscardi S. (2020). Kawasaki-like multisystem inflammatory syndrome in children during the COVID-19 pandemic in Paris, France: Prospective observational study. BMJ.

[5] Whittaker E., Bamford A., Kenny J., Kaforou M., Jones C., Shah P., Ramnarayan P., Fraisse A., Miller O., Davies P. (2020). Clinical Characteristics of 58 Children with a Pediatric Inflammatory Multisystem Syndrome Temporally Associated with SARS-CoV-2. JAMA.

[6] Singh-Grewal D., Lucas R., Macartney K., Cheng A.C., Wood N., Ostring G., Britton P., Crawford N., Burgner D. (2020). Update on the COVID-19-associated inflammatory syndrome in children and adolescents; paediatric inflammatory multisystem syndrome-temporally associated with SARS-CoV-2. J. Paediatr. Child Health.

[7] Verdoni L., Mazza A., Gervasoni A., Martelli L., Ruggeri M., Ciuffreda M., Bonanomi E., D’Antiga L. (2020). An outbreak of severe Kawasaki-like disease at the Italian epicentre of the SARS-CoV-2 epidemic: An observational cohort study. Lancet.

[8] World Health Organization (WHO) (2020). Multisystem Inflammatory Syndrome in Children and Adolescents Temporally Related to COVID-19.

[9] Dove M.L., Jaggi P., Kelleman M., Abuali M., Ang J.Y., Ballan W., Basu S.K., Campbell M.J., Chikkabyrappa S.M., Choueiter N.F. (2020). Multisystem Inflammatory Syndrome in Children: Survey of Protocols for Early Hospital Evaluation and Management. J. Pediatr..

[10] Sachdeva V., Roy A., Bharadvaja N. (2020). Current Prospects of Nutraceuticals: A Review. Curr. Pharm. Biotechnol..

[11] Ronis M.J., Pedersen K.B., Watt J. (2018). Adverse Effects of Nutraceuticals and Dietary Supplements. Annu. Rev. Pharmacol. Toxicol..

[12] Aronson J.K. (2016). Defining ‘nutraceuticals’: Neither nutritious nor pharmaceutical. Br. J. Clin. Pharmacol..

[13] Akhtar S., Das J.K., Ismail T., Wahid M., Saeed W., Bhutta Z.A. (2020). Nutritional perspectives for the prevention and mitigation of COVID-19. Nutr. Rev..

[14] Sikander M., Malik S., Rodriguez A., Yallapu M.M., Narula A.S., Satapathy S.K., Dhevan V., Chauhan S.C., Jaggi M. (2020). Role of Nutraceuticals in COVID-19 Mediated Liver Dysfunction. Molecules.

[15] (2020). Paediatric Intensive Care Society PICS Statement: Increased Number of Reported Cases of Novel Presentation of Multisystem Inflammatory Disease.

[16] Vogel T.P., Top K.A., Karatzios C., Hilmers D.C., Tapia L.I., Moceri P., Giovannini-Chami L., Wood N., Chandler R.E., Klein N.P. (2021). Multisystem inflammatory syndrome in children and adults (MIS-C/A): Case definition & guidelines for data collection, analysis, and presentation of immunization safety data. Vaccine.

[17] Carter M.J., Shankar-Hari M., Tibby S.M. (2020). Paediatric Inflammatory Multisystem Syndrome Temporally-Associated with SARS-CoV-2 Infection: An Overview. Intensiv. Care Med..

[18] Centers for Disease Control and Prevention (CDC) (2020). Health Alert Network Multisystem Inflammatory Syndrome in Children (MIS-C) Associated with Coronavirus Disease 2019 (COVID-19).

[19] Kabeerdoss J., Pilania R.K., Karkhele R., Kumar T.S., Danda D., Singh S. (2020). Severe COVID-19, multisystem inflammatory syndrome in children, and Kawasaki disease: Immunological mechanisms, clinical manifestations and management. Rheumatol. Int..

[20] (2022). American Academy of Pediatrics Multisystem Inflammatory Syndrome in Children (MIS-C) Interim Guidance.

[21] Henderson L.A., Canna S.W., Friedman K.G., Gorelik M., Lapidus S.K., Bassiri H., Behrens E.M., Kernan K.F., Schulert G.S., Seo P. (2022). American College of Rheumatology Clinical Guidance for Multisystem Inflammatory Syndrome in Children Associated with SARS–CoV-2 and Hyperinflammation in Pediatric COVID-19: Version 3. Arthritis Rheumatol..

[22] Feldstein L.R., Rose E.B., Horwitz S.M., Collins J.P., Newhams M.M., Son M.B.F., Newburger J.W., Kleinman L.C., Heidemann S.M., Martin A.A. (2020). Multisystem Inflammatory Syndrome in U.S. Children and Adolescents. N. Engl. J. Med..

[23] Zhang Q.-Y., Xu B.-W., Du J.-B. (2021). Similarities and differences between multiple inflammatory syndrome in children associated with COVID-19 and Kawasaki disease: Clinical presentations, diagnosis, and treatment. World J. Pediatr..

[24] Feldstein L.R., Tenforde M.W., Friedman K.G., Newhams M., Rose E.B., Dapul H., Soma V.L., Maddux A.B., Mourani P.M., Bowens C. (2021). Characteristics and Outcomes of US Children and Adolescents with Multisystem Inflammatory Syndrome in Children (MIS-C) Compared with Severe Acute COVID-19. JAMA.

[25] Jain S., Sen S., Lakshmivenkateshiah S., Bobhate P., Venkatesh S., Udani S., Shobhavat L., Andankar P., Karande T., Kulkarni S. (2020). Multisystem Inflammatory Syndrome in Children with COVID-19 in Mumbai, India. Indian Pediatr..

[26] Williams V., Dash N., Suthar R., Mohandoss V., Jaiswal N., Kavitha T., Nallasamy K., Angurana S.K. (2020). Clinicolaboratory Profile, Treatment, Intensive Care Needs, and Outcome of Pediatric Inflammatory Multisystem Syndrome Temporally Associated with SARS-CoV-2: A Systematic Review and Meta-analysis. J. Pediatr. Intensiv. Care.

[27] Ciftdogan D.Y., Keles Y.E., Karbuz A., Cetin B.S., Bozdemir S.E., Kadayifci E.K., Akcan O.M., Ozer A., Erat T., Sutcu M. (2022). Multisystem inflammatory syndrome in children associated with COVID -19 in 101 cases from Turkey (Turk-MISC study). J. Paediatr. Child Health.

[28] Yonker L.M., Shen K., Kinane T.B. (2020). Lessons unfolding from pediatric cases of COVID-19 disease caused by SARS-CoV-2 infection. Pediatr. Pulmonol..

[29] Pollock N.R., Savage T.J., Wardell H., Lee R.A., Mathew A., Stengelin M., Sigal G.B. (2021). Correlation of SARS-CoV-2 Nucleocapsid Antigen and RNA Concentrations in Nasopharyngeal Samples from Children and Adults Using an Ultrasensitive and Quantitative Antigen Assay. J. Clin. Microbiol..

[30] Sigal G.B., Novak T., Mathew A., Chou J., Zhang Y., Manjula N., Bathala P., Joe J., Padmanabhan N., Romero D. (2022). Measurement of SARS-CoV-2 Antigens in Plasma of Pediatric Patients with Acute COVID-19 or Multisystem Inflammatory Syndrome in Children (MIS-C) Using an Ultrasensitive and Quantitative Immunoassay. Clin. Infect. Dis..

[31] Madjid M., Safavi-Naeini P., Solomon S.D., Vardeny O. (2020). Potential effects of coronaviruses on the cardiovascular system: A review. JAMA Cardiol..

[32] Felsenstein S., Duong P., Lane S., Jones C., Pain C.E., Hedrich C.M. (2021). Cardiac pathology and outcomes vary between Kawasaki disease and PIMS-TS. Clin. Immunol..

[33] Liu K., Yu J., Song G. (2022). Global Myocardial Strain in Multisystem Inflammatory Syndrome in Children, Kawasaki Disease, and Healthy Children: A Network Meta-Analysis. Front. Pediatr..

[34] Rodriguez-Gonzalez M., Castellano-Martinez A., Cascales-Poyatos H.M., Perez-Reviriego A.A. (2020). Cardiovascular impact of COVID-19 with a focus on children: A systematic review. World J. Clin. Cases.

[35] Blatz A.M., Randolph A.G. (2022). Severe COVID-19 and Multisystem Inflammatory Syndrome in Children in Children and Adolescents. Crit. Care Clin..

[36] Zhao Y., Patel J., Huang Y., Yin L., Tang L. (2021). Cardiac markers of multisystem inflammatory syndrome in children (MIS-C) in COVID-19 patients: A meta-analysis. Am. J. Emerg. Med..

[37] Lee P.Y., Day-Lewis M., Henderson L.A., Friedman K.G., Lo J., Roberts J.E., Lo M.S., Platt C.D., Chou J., Hoyt K.J. (2020). Distinct clinical and immunological features of SARS–CoV-2–induced multisystem inflammatory syndrome in children. J. Clin. Investig..

[38] Cheung E.W., Zachariah P., Gorelik M., Boneparth A., Kernie S., Orange J.S., Milner J.D. (2020). Multisystem Inflammatory Syndrome Related to COVID-19 in Previously Healthy Children and Adolescents in New York City. JAMA.

[39] Valverde I., Singh Y., Sanchez-De-Toledo J., Theocharis P., Chikermane A., Di Filippo S., Kuciñska B., Mannarino S., Tamariz-Martel A., Gutierrez-Larraya F. (2021). Acute Cardiovascular Manifestations in 286 Children with Multisystem Inflammatory Syndrome Associated with COVID-19 Infection in Europe. Circulation.

[40] Vella L.A., Rowley A.H. (2021). Current Insights into the Pathophysiology of Multisystem Inflammatory Syndrome in Children. Curr. Pediatr. Rep..

[41] Lee K.H., Li H., Lee M.H., Park S.J., Kim J.S., Han Y.J., Cho K., Ha B., Kim S.J., Jacob L. (2022). Clinical Characteristics and Treatments of Multi-System Inflammatory Syndrome in Children: A Systematic Review. Eur. Rev. Med. Pharmacol. Sci..

[42] Gurlevik S.L., Ozsurekci Y., Sağ E., Oygar P.D., Kesici S., Akca K., Cuceoglu M.K., Basaran O., Göncü S., Karakaya J. (2022). The difference of the inflammatory milieu in MIS-C and severe COVID-19. Pediatr. Res..

[43] Schmitz A., Wood K.E., Badheka M.A., Burghardt M.E., Wendt M.L., Sharathkumar M.A., Koestner B. (2022). NT-proBNP Levels Following IVIG Treatment of Multisystem Inflammatory Syndrome in Children. Hosp. Pediatr..

[44] Bohn M.K., Yousef P., Steele S., Sepiashvili L., Adeli K. (2021). MultiInflammatory Syndrome in Children: A View into Immune Pathogenesis from a Laboratory Perspective. J. Appl. Lab. Med..

[45] Anderson E.M., Diorio C., Goodwin E.C., McNerney K.O., Weirick M.E., Gouma S., Bolton M.J., Arevalo C.P., Chase J., Hicks P. (2020). Severe Acute Respiratory Syndrome-Coronavirus-2 (SARS-CoV-2) Antibody Responses in Children with Multisystem Inflammatory Syndrome in Children (MIS-C) and Mild and Severe Coronavirus Disease 2019 (COVID-19). J. Pediatr. Infect. Dis. Soc..

[46] Gruber C.N., Patel R.S., Trachtman R., Lepow L., Amanat F., Krammer F., Wilson K.M., Onel K., Geanon D., Tuballes K. (2020). Mapping Systemic Inflammation and Antibody Responses in Multisystem Inflammatory Syndrome in Children (MIS-C). Cell.

[47] Abrams J.Y., Oster M.E., Godfred-Cato S.E., Bryant B., Datta S.D., Campbell A.P., Leung J.W., Tsang C.A., Pierce T.J., Kennedy J.L. (2021). Factors linked to severe outcomes in multisystem inflammatory syndrome in children (MIS-C) in the USA: A retrospective surveillance study. Lancet Child Adolesc. Health.

[48] Rodriguez-Smith J.J., Verweyen E.L., Clay G.M., Esteban Y.M., de Loizaga S.R., Baker E.J., Do T., Dhakal S., Lang S.M., Grom A.A. (2021). Inflammatory biomarkers in COVID-19-associated multisystem inflammatory syndrome in children, Kawasaki disease, and macrophage activation syndrome: A cohort study. Lancet Rheumatol..

[49] Kostik M.M., Bregel L.V., Avrusin I.S., Dondurei E.A., Matyunova A.E., Efremova O.S., Isupova E.A., Kornishina T.L., Masalova V.V., Snegireva L.S. (2021). Distinguishing Between Multisystem Inflammatory Syndrome, Associated with COVID-19 in Children and the Kawasaki Disease: Development of Preliminary Criteria Based on the Data of the Retrospective Multicenter Cohort Study. Front. Pediatr..

[50] Belay E.D., Abrams J., Oster M.E., Giovanni J., Pierce T., Meng L., Prezzato E., Balachandran N., Openshaw J.J., Rosen H.E. (2021). Trends in Geographic and Temporal Distribution of US Children with Multisystem Inflammatory Syndrome During the COVID-19 Pandemic. JAMA Pediatr..

[51] Ganguly M., Nandi A., Banerjee P., Gupta P., Sarkar S.D., Basu S., Pal P. (2021). A comparative study of IL-6, CRP and NT-proBNP levels in post-COVID multisystem inflammatory syndrome in children (MISC) and Kawasaki disease patients. Int. J. Rheum. Dis..

[52] Carter M.J., Fish M., Jennings A., Doores K.J., Wellman P., Seow J., Acors S., Graham C., Timms E., Kenny J. (2020). Peripheral immunophenotypes in children with multisystem inflammatory syndrome associated with SARS-CoV-2 infection. Nat. Med..

[53] Venkataraman A., Kumar N.P., Hanna L.E., Putlibai S., Karthick M., Rajamanikam A., Sadasivam K., Sundaram B., Babu S. (2021). Plasma biomarker profiling of PIMS-TS, COVID-19 and SARS-CoV2 seropositive children—A cross-sectional observational study from southern India. eBioMedicine.

[54] Zhao Y., Yin L., Patel J., Tang L., Huang Y. (2021). The inflammatory markers of multisystem inflammatory syndrome in children (MIS-C) and adolescents associated with COVID-19: A meta-analysis. J. Med. Virol..

[55] Abo-Haded H.M., Alshengeti A.M., Alawfi A.D., Khoshhal S.Q., Al-Harbi K.M., Allugmani M.D., El-Agamy D.S. (2022). Cytokine Profiling among Children with Multisystem Inflammatory Syndrome versus Simple COVID-19 Infection: A Study from Northwest Saudi Arabia. Biology.

[56] Caldarale F., Giacomelli M., Garrafa E., Tamassia N., Morreale A., Poli P., Timpano S., Baresi G., Zunica F., Cattalini M. (2021). Plasmacytoid Dendritic Cells Depletion and Elevation of IFN-γ Dependent Chemokines CXCL9 and CXCL10 in Children with Multisystem Inflammatory Syndrome. Front. Immunol..

[57] Kumar N.P., Venkataraman A., Nancy A., Moideen K., Varadarjan P., Selladurai E., Sangaralingam T., Selvam R., Thimmaiah A., Natarajan S. (2022). Enhanced Severe Acute Respiratory Syndrome Coronavirus 2 Antigen–Specific Systemic Immune Responses in Multisystem Inflammatory Syndrome in Children and Reversal After Recovery. J. Infect. Dis..

[58] Akindele N.P., Kouo T., Karaba A.H., Gordon O., Fenstermacher K.Z.J., Beaudry J., Rubens J.H., Atik C.C., Zhou W., Ji H. (2021). Distinct Cytokine and Chemokine Dysregulation in Hospitalized Children with Acute Coronavirus Disease 2019 and Multisystem Inflammatory Syndrome with Similar Levels of Nasopharyngeal Severe Acute Respiratory Syndrome Coronavirus 2 Shedding. J. Infect. Dis..

[59] Ramaswamy A., Brodsky N.N., Sumida T.S., Comi M., Asashima H., Hoehn K.B., Li N., Liu Y., Shah A., Ravindra N.G. (2021). Immune dysregulation and autoreactivity correlate with disease severity in SARS-CoV-2-associated multisystem inflammatory syndrome in children. Immunity.

[60] Vella L.A., Giles J.R., Baxter A.E., Oldridge D.A., Diorio C., Kuri-Cervantes L., Alanio C., Pampena M.B., Wu J.E., Chen Z. (2021). Deep immune profiling of MIS-C demonstrates marked but transient immune activation compared with adult and pediatric COVID-19. Sci. Immunol..

[61] Mazzoni A., Salvati L., Maggi L., Annunziato F., Cosmi L. (2021). Hallmarks of immune response in COVID-19: Exploring dysregulation and exhaustion. Semin. Immunol..

[62] Bordoni V., Sacchi A., Cimini E., Notari S., Grassi G., Tartaglia E., Casetti R., Giancola M.L., Bevilacqua N., Maeurer M. (2020). An Inflammatory Profile Correlates with Decreased Frequency of Cytotoxic Cells in Coronavirus Disease 2019. Clin. Infect. Dis..

[63] Zhou Z., He H., Wang K., Shi X., Wang Y., Su Y., Wang Y., Li D., Liu W., Zhang Y. (2020). Granzyme A from cytotoxic lymphocytes cleaves GSDMB to trigger pyroptosis in target cells. Science.

[64] Zhou C., Zhao Y., Wang X., Huang Y., Tang X., Tang L. (2021). Laboratory parameters between multisystem inflammatory syndrome in children and Kawasaki disease. Pediatr. Pulmonol..

[65] Hoang A., Chorath K., Moreira A., Evans M., Burmeister-Morton F., Burmeister F., Naqvi R., Petershack M., Moreira A. (2020). COVID-19 in 7780 pediatric patients: A systematic review. eClinicalMedicine.

[66] Stasiak A., Perdas E., Smolewska E. (2022). Risk factors of a severe course of pediatric multi-system inflammatory syndrome temporally associated with COVID-19. Eur. J. Pediatr..

[67] Yonker L.M., Gilboa T., Ogata A.F., Senussi Y., Lazarovits R., Boribong B.P., Bartsch Y.C., Loiselle M., Rivas M.N., Porritt R.A. (2021). Multisystem inflammatory syndrome in children is driven by zonulin-dependent loss of gut mucosal barrier. J. Clin. Investig..

[68] McCafferty C., Cai T., Borgel D., Lasne D., Renolleau S., Vedrenne-Cloquet M., Bonnet D., Wu J., Zaw T., Bhatnagar A. (2022). Pathophysiological pathway differences in children who present with COVID-19 ARDS compared to COVID -19 induced MIS-C. Nat. Commun..

[69] Huang J.J., Gaines S.B., Amezcua M.L., Lubell T.R., Dayan P.S., Dale M., Boneparth A.D., Hicar M.D., Winchester R., Gorelik M. (2022). Upregulation of type 1 conventional dendritic cells implicates antigen cross-presentation in multisystem inflammatory syndrome. J. Allergy Clin. Immunol..

[70] Ciftdogan D.Y., Keles Y.E., Cetin B.S., Karabulut N.D., Emiroglu M., Bagci Z., Buyukcam A., Erdeniz E.H., Arga G., Yesil E. (2022). COVID-19 associated multisystemic inflammatory syndrome in 614 children with and without overlap with Kawasaki disease-Turk MIS-C study group. Eur. J. Pediatr..

[71] Yakut N., Yuksel E., Algul M., Armut M., Sahin B., Karagoz G., Yakut K., Kilinc A., Tanidir I.C. (2022). Comparison of clinical and laboratory features in coronavirus disease 2019 and pediatric multisystem inflammatory syndrome patients. Pediatr. Int..

[72] Beckmann N.D., Comella P.H., Cheng E., Lepow L., Beckmann A.G., Tyler S.R., Mouskas K., Simons N.W., Hoffman G.E., Francoeur N.J. (2021). Downregulation of exhausted cytotoxic T cells in gene expression networks of multisystem inflammatory syndrome in children. Nat. Commun..

[73] Morita A., Hosaka S., Imagawa K., Ishiodori T., Nozaki Y., Murakami T., Takada H. (2022). Time course of peripheral immunophenotypes of multisystem inflammatory syndrome in children. Clin. Immunol..

[74] Hsieh L.-E., Song J., Grifoni A., Shimizu C., Tremoulet A.H., Dummer K.B., Burns J.C., Sette A., Franco A. (2022). T Cells in Multisystem Inflammatory Syndrome in Children (MIS-C) Have a Predominant CD4+ T Helper Response to SARS-CoV-2 Peptides and Numerous Virus-Specific CD4− CD8− Double-Negative T Cells. Int. J. Mol. Sci..

[75] Talarico L.B., Toledano A., Contrini M.M., Torrado L.E., Martínez M.P., Gaillard M.I., Caratozzolo A., Byrne A.B., Bonnin F.A., Tineo M.S. (2022). Distinct Immune Phenotypes and Cytokine Profiles in Children with Differing Severity of COVID-19. Pediatr. Infect. Dis. J..

[76] Singh V., Obregon-Perko V., Lapp S.A., Horner A.M., Brooks A., Macoy L., Hussaini L., Lu A., Gibson T., Silvestri G. (2022). Limited induction of SARS-CoV-2–specific T cell responses in children with multisystem inflammatory syndrome compared with COVID-19. JCI Insight.

[77] Moreews M., Le Gouge K., Khaldi-Plassart S., Pescarmona R., Mathieu A.-L., Malcus C., Djebali S., Bellomo A., Dauwalder O., Perret M. (2021). Polyclonal expansion of TCR Vb 21.3 ^+^ CD4^+^ and CD8^+^ T cells is a hallmark of multisystem inflammatory syndrome in children. Sci. Immunol..

[78] Zheng M., Gao Y., Wang G., Song G., Liu S., Sun D., Xu Y., Tian Z. (2020). Functional exhaustion of antiviral lymphocytes in COVID-19 patients. Cell. Mol. Immunol..

[79] Asl K.D., Mazloumi Z., Majidi G., Kalarestaghi H., Sabetkam S., Rafat A. (2022). NK cell dysfunction is linked with disease severity in SARS-CoV-2 patients. Cell Biochem. Funct..

[80] Mukund K., Nayak P., Ashokkumar C., Rao S., Almeda J., Betancourt-Garcia M.M., Sindhi R., Subramaniam S. (2021). Immune Response in Severe and Non-Severe Coronavirus Disease 2019 (COVID-19) Infection: A Mechanistic Landscape. Front. Immunol..

[81] Chau A.S., Weber A.G., Maria N.I., Narain S., Liu A., Hajizadeh N., Malhotra P., Bloom O., Marder G., Kaplan B. (2021). The Longitudinal Immune Response to Coronavirus Disease 2019: Chasing the Cytokine Storm. Arthritis Rheumatol..

[82] Nagelkerke S.Q., Kuijpers T.W. (2015). Immunomodulation by IVIg and the Role of Fc-Gamma Receptors: Classic Mechanisms of Action after All?. Front. Immunol..

[83] Mori M., Miyamae T., Imagawa T., Katakura S., Kimura K., Yokota S. (2004). Meta-analysis of the results of intravenous gamma globulin treatment of coronary artery lesions in Kawasaki disease. Mod. Rheumatol..

[84] Terai M., Shulman S.T. (1997). Prevalence of coronary artery abnormalities in Kawasaki disease is highly dependent on gamma globulin dose but independent of salicylate dose. J. Pediatr..

[85] Burns J.C., Franco A. (2015). The immunomodulatory effects of intravenous immunoglobulin therapy in Kawasaki disease. Expert Rev. Clin. Immunol..

[86] McCrindle B.W., Rowley A.H., Newburger J.W., Burns J.C., Bolger A.F., Gewitz M., Baker A.L., Jackson M.A., Takahashi M., Shah P.B. (2017). Diagnosis, Treatment, and Long-Term Management of Kawasaki Disease: A Scientific Statement for Health Professionals from the American Heart Association. Circulation.

[87] Belhadjer Z., Auriau J., Méot M., Oualha M., Renolleau S., Houyel L., Bonnet D. (2020). Addition of Corticosteroids to Immunoglobulins Is Associated with Recovery of Cardiac Function in Multi-Inflammatory Syndrome in Children. Circulation.

[88] Algarni A.S., Alamri N.M., Khayat N.Z., Alabdali R.A., Alsubhi R.S., Alghamdi S.H. (2022). Clinical practice guidelines in multisystem inflammatory syndrome (MIS-C) related to COVID-19: A critical review and recommendations. World J. Pediatr..

[89] McArdle A.J., Vito O., Patel H., Seaby E.G., Shah P., Wilson C., Broderick C., Nijman R., Tremoulet A.H., Munblit D. (2021). Treatment of Multisystem Inflammatory Syndrome in Children. N. Engl. J. Med..

[90] Ouldali N., Toubiana J., Antona D., Javouhey E., Madhi F., Lorrot M., Léger P.-L., Galeotti C., Claude C., Wiedemann A. (2021). Association of Intravenous Immunoglobulins Plus Methylprednisolone vs Immunoglobulins Alone with Course of Fever in Multisystem Inflammatory Syndrome in Children. JAMA.

[91] Ciccarelli G.P., Bruzzese E., Asile G., Vassallo E., Pierri L., De Lucia V., Guarino A., Vecchio A.L. (2021). Bradycardia associated with Multisystem Inflammatory Syndrome in Children with COVID-19: A case series. Eur. Heart J.-Case Rep..

[92] Cui H.-Y., Hu C.-P. (2022). Recent Research on the Application of Biologics in the Treatment of Multisystem Inflammatory Syndrome in Children after SARS-CoV-2 Infection. CJCP.

[93] Cole L.D., Osborne C.M., Silveira L.J., Rao S., Lockwood J.M., Kunkel M.J., MacBrayne C.E., Heizer H.R., Anderson M.S., Jone P.-N. (2021). IVIG Compared with IVIG Plus Infliximab in Multisystem Inflammatory Syndrome in Children. Pediatrics.

[94] Thom K., Kahl B., Wagner T., van Egmond-Fröhlich A., Krainz M., Frischer T., Leeb I., Schuster C., Ehringer-Schetitska D., Minkov M. (2022). SARS-CoV-2 Associated Pediatric Inflammatory Multisystem Syndrome with a High Prevalence of Myocarditis—A Multicenter Evaluation of Clinical and Laboratory Characteristics, Treatment and Outcome. Front. Pediatr..

[95] Sözeri B., Çağlayan Ş., Atasayan V., Ulu K., Coşkuner T., Akbay P., Akkuş C.H., Atay G., Salı E., Karacan M. (2021). The clinical course and short-term health outcomes of multisystem inflammatory syndrome in children in the single pediatric rheumatology center. Postgrad. Med..

[96] Yamaguchi Y., Takasawa K., Irabu H., Hiratoko K., Ichigi Y., Hirata K., Tamura Y., Murakoshi M., Yamashita M., Nakatani H. (2022). Infliximab treatment for refractory COVID-19-associated multisystem inflammatory syndrome in a Japanese child. J. Infect. Chemother..

[97] Kalra E.K. (2003). Nutraceutical—Definition and introduction. AAPS Pharm. Sci..

[98] Kemppainen L.M., Kemppainen T., Reippainen J.A., Salmenniemi S.T., Vuolanto P. (2017). Use of complementary and alternative medicine in Europe: Health-related and sociodemographic determinants. Scand. J. Public Health.

[99] Tangkiatkumjai M., Boardman H., Walker D.-M. (2020). Potential factors that influence usage of complementary and alternative medicine worldwide: A systematic review. BMC Complement. Med. Ther..

[100] Teoh S.L., Ngorsuraches S., Lai N.M., Bangpan M., Chaiyakunapruk N. (2019). Factors affecting consumers’ decisions on the use of nutraceuticals: A systematic review. Int. J. Food Sci. Nutr..

[101] Menon A., Sawant M., Mishra S., Bhatia P., Rathod S. (2021). Awareness, Perception and Usage of Nutraceuticals in Indian Society. Int. J. Sci. Res. Sci. Technol..

[102] Bukic J., Kuzmanic B., Rusic D., Portolan M., Mihanovic A., Perisin A.S., Leskur D., Petric A., Bozic J., Tomic S. (2021). Community pharmacists’ use, perception and knowledge on dietary supplements: A cross sectional study. Pharm. Pract. (Granada).

[103] Cruz C.S.D., Kang M.-J. (2017). Mitochondrial dysfunction and damage associated molecular patterns (DAMPs) in chronic inflammatory diseases. Mitochondrion.

[104] Volp A.C.P. (2015). Hepatic Inflammatory Biomarkers and Its Link with Obesity and Chronic Diseases. Nutr. Hosp..

[105] Tsoupras A., Lordan R., Zabetakis I. (2018). Inflammation, not Cholesterol, Is a Cause of Chronic Disease. Nutrients.

[106] Cheresh P., Kim S.-J., Tulasiram S., Kamp D.W. (2013). Oxidative stress and pulmonary fibrosis. Biochim. et Biophys. Acta (BBA)-Mol. Basis Dis..

[107] Luc K., Schramm-Luc A., Guzik T.J., Mikolajczyk T.P. (2019). Oxidative stress and inflammatory markers in prediabetes and dia-betes. J. Physiol. Pharmacol..

[108] Peoples J.N., Saraf A., Ghazal N., Pham T.T., Kwong J.Q. (2019). Mitochondrial dysfunction and oxidative stress in heart disease. Exp. Mol. Med..

[109] Valavanidis A., Vlachogianni T., Fiotakis K., Loridas S. (2013). Pulmonary Oxidative Stress, Inflammation and Cancer: Respirable Particulate Matter, Fibrous Dusts and Ozone as Major Causes of Lung Carcinogenesis through Reactive Oxygen Species Mechanisms. Int. J. Environ. Res. Public Health.

[110] Calaça C.E. (2002). Medicines and medical plants in the tropics: On the development of Western science of pharmacy. Hist Cienc. Saude Manguinhos.

[111] Calixto J. (2000). Efficacy, safety, quality control, marketing and regulatory guidelines for herbal medicines (phytotherapeutic agents). Braz. J. Med. Biol. Res..

[112] Shu Y.-Z. (1998). Recent Natural Products Based Drug Development: A Pharmaceutical Industry Perspective. J. Nat. Prod..

[113] Sahebnasagh A., Saghafi F., Avan R., Khoshi A., Khataminia M., Safdari M., Habtemariam S., Ghaleno H.R., Nabavi S.M. (2020). The prophylaxis and treatment potential of supplements for COVID-19. Eur. J. Pharmacol..

[114] Andersen C.J. (2015). Bioactive Egg Components and Inflammation. Nutrients.

[115] Diniz do Nascimento L., De Moraes A.A.B., Da Costa K.S., Galúcio J.M.P., Taube P.S., Costa C.M.L., Cruz J.N., Andrade E.H.D.A., De Faria L.J.G. (2020). Bioactive Natural Compounds and Antioxidant Activity of Essential Oils from Spice Plants: New Findings and Potential Applications. Biomolecules.

[116] Chen T.-R., Wei L.-H., Guan X.-Q., Huang C., Liu Z.-Y., Wang F.-J., Hou J., Jin Q., Liu Y.-F., Wen P.-H. (2019). Biflavones from Ginkgo biloba as inhibitors of human thrombin. Bioorg. Chem..

[117] Shanmugam K.R., Shanmugam B., Subbaiah G.V., Ravi S., Reddy K.S. (2021). Medicinal Plants and Bioactive Compounds for Diabetes Management: Important Advances in Drug Discovery. Curr. Pharm. Des..

[118] Li Y., Tan Y.-F., Wei N., Zhang J.-Q. (2016). Diuretic and Anti-Diuretic Bioactivity Differences of the Seed and Shell Extracts of Alpinia Oxyphylla Fruit. Afr. J. Tradit. Complement. Altern. Med..

[119] Estrada-Luna D., Ortiz-Rodriguez M.A., Medina-Briseño L., Carreón-Torres E., Izquierdo-Vega J.A., Sharma A., Cancino-Díaz J.C., Pérez-Méndez O., Belefant-Miller H., Betanzos-Cabrera G. (2018). Current Therapies Focused on High-Density Lipoproteins Associated with Cardiovascular Disease. Molecules.

[120] Infusino F., Marazzato M., Mancone M., Fedele F., Mastroianni C.M., Severino P., Ceccarelli G., Santinelli L., Cavarretta E., Marullo A.G.M. (2020). Diet Supplementation, Probiotics, and Nutraceuticals in SARS-CoV-2 Infection: A Scoping Review. Nutrients.

[121] Deng Y.-B., Xiang H.-J., Chang Q., Li C.-L. (2002). Evaluation by High-Resolution Ultrasonography of Endothelial Function in Brachial Artery After Kawasaki Disease and the Effects of Intravenous Administration of Vitamin C. Circ. J..

[122] Hassaniazad M., Eftekhar E., Inchehsablagh B.R., Kamali H., Tousi A., Jaafari M.R., Rafat M., Fathalipour M., Nikoofal-Sahlabadi S., Gouklani H. (2021). A triple-blind, placebo-controlled, randomized clinical trial to evaluate the effect of curcumin-containing nanomicelles on cellular immune responses subtypes and clinical outcome in COVID-19 patients. Phytotherapy Res..

[123] Shafie E.H., Taheri F., Alijani N., Okhovvat A.R., Goudarzi R., Borumandnia N., Aghaghazvini L., Rezayat S.M., Jamalimoghadamsiahkali S., Hosseinzadeh-Attar M.J. (2022). Effect of nanocurcumin supplementation on the severity of symptoms and length of hospital stay in patients with COVID-19: A randomized double-blind placebo-controlled trial. Phytotherapy Res..

[124] Askari G., Sahebkar A., Soleimani D., Mahdavi A., Rafiee S., Majeed M., Khorvash F., Iraj B., Elyasi M., Rouhani M.H. (2022). The efficacy of curcumin-piperine co-supplementation on clinical symptoms, duration, severity, and inflammatory factors in COVID-19 outpatients: A randomized double-blind, placebo-controlled trial. Trials.

[125] Sedighiyan M., Abdollahi H., Karimi E., Badeli M., Erfanian R., Raeesi S., Hashemi R., Vahabi Z., Asanjarani B., Mansouri F. (2021). Omega-3 polyunsaturated fatty acids supplementation improve clinical symptoms in patients with COVID-19: A randomised clinical trial. Int. J. Clin. Pract..

[126] Doaei S., Gholami S., Rastgoo S., Gholamalizadeh M., Bourbour F., Bagheri S.E., Samipoor F., Akbari M.E., Shadnoush M., Ghorat F. (2021). The effect of omega-3 fatty acid supplementation on clinical and biochemical parameters of critically ill patients with COVID-19: A randomized clinical trial. J. Transl. Med..

[127] Fessler S.N., Liu L., Chang Y., Yip T., Johnston C.S. (2022). Palmitoylethanolamide Reduces Proinflammatory Markers in Unvaccinated Adults Recently Diagnosed with COVID-19: A Randomized Controlled Trial. J. Nutr..

[128] Di Pierro F., Derosa G., Maffioli P., Bertuccioli A., Togni S., Riva A., Allegrini P., Khan A., Khan S., Khan B.A. (2021). Possible Therapeutic Effects of Adjuvant Quercetin Supplementation Against Early-Stage COVID-19 Infection: A Prospective, Randomized, Controlled, and Open-Label Study. Int. J. Gen. Med..

[129] Di Pierro F., Iqtadar S., Khan A., Mumtaz S.U., Chaudhry M.M., Bertuccioli A., Derosa G., Maffioli P., Togni S., Riva A. (2021). Potential Clinical Benefits of Quercetin in the Early Stage of COVID-19: Results of a Second, Pilot, Randomized, Controlled and Open-Label Clinical Trial. Int. J. Gen. Med..

[130] Rohani M.R., Mozaffar H., Mesri M., Shokri M., Delaney D., Karimy M. (2022). Evaluation and comparison of the effect of vitamin A supplementation with standard therapies in the treatment of patients with COVID-19. East. Mediterr. Health J..

[131] Beigmohammadi M.T., Bitarafan S., Hoseindokht A., Abdollahi A., Amoozadeh L., Soltani D. (2021). The effect of supplementation with vitamins A, B, C, D, and E on disease severity and inflammatory responses in patients with COVID-19: A randomized clinical trial. Trials.

[132] Majidi N., Rabbani F., Gholami S., Gholamalizadeh M., BourBour F., Rastgoo S., Hajipour A., Shadnoosh M., Akbari M.E., Bahar B. (2021). The Effect of Vitamin C on Pathological Parameters and Survival Duration of Critically Ill Coronavirus Disease 2019 Patients: A Randomized Clinical Trial. Front. Immunol..

[133] JamaliMoghadamSiahkali S., Zarezade B., Koolaji S., SeyedAlinaghi S., Zendehdel A., Tabarestani M., Moghadam E.S., Abbasian L., Manshadi S.A.D., Salehi M. (2021). Safety and effectiveness of high-dose vitamin C in patients with COVID-19: A randomized open-label clinical trial. Eur. J. Med. Res..

[134] Mariani J., Antonietti L., Tajer C., Ferder L., Inserra F., Cunto M.S., Brosio D., Ross F., Zylberman M., López D.E. (2022). High-dose vitamin D versus placebo to prevent complications in COVID-19 patients: Multicentre randomized controlled clinical trial. PLoS ONE.

[135] Annweiler C., Beaudenon M., Gautier J., Gonsard J., Boucher S., Chapelet G., Darsonval A., Fougère B., Guérin O., Houvet M. (2022). High-Dose versus Standard-Dose Vitamin D Supplementation in Older Adults with COVID-19 (COVIT-TRIAL): A Multicenter, Open-Label, Randomized Controlled Superiority Trial. PLoS Med..

[136] Ling S.F., Broad E., Murphy R., Pappachan J.M., Pardesi-Newton S., Kong M.F., Jude E.B. (2020). High-Dose Cholecalciferol Booster Therapy is Associated with a Reduced Risk of Mortality in Patients with COVID-19: A Cross-Sectional Multi-Centre Observational Study. Nutrients.

[137] Fernandes A.L., Murai I.H., Reis B.Z., Sales L.P., Santos M.D., Pinto A.J., Goessler K.F., Duran C.S.C., Silva C.B.R., Franco A.S. (2022). Effect of a single high dose of vitamin D3 on cytokines, chemokines, and growth factor in patients with moderate to severe COVID-19. Am. J. Clin. Nutr..

[138] Murai I.H., Fernandes A.L., Sales L.P., Pinto A.J., Goessler K.F., Duran C.S.C., Silva C.B.R., Franco A.S., Macedo M.B., Dalmolin H.H.H. (2021). Effect of a Single High Dose of Vitamin D_3_ on Hospital Length of Stay in Patients with Moderate to Severe COVID-19: A Randomized Clinical Trial. JAMA.

[139] Annweiler C., Beaudenon M., Simon R., Guenet M., Otekpo M., Célarier T., Gautier J. (2021). Vitamin D supplementation prior to or during COVID-19 associated with better 3-month survival in geriatric patients: Extension phase of the GERIA-COVID study. J. Steroid Biochem. Mol. Biol..

[140] Karonova T.L., Chernikova A.T., Golovatyuk K.A., Bykova E.S., Grant W.B., Kalinina O.V., Grineva E.N., Shlyakhto E.V. (2022). Vitamin D Intake May Reduce SARS-CoV-2 Infection Morbidity in Health Care Workers. Nutrients.

[141] Karonova T.L., Golovatyuk K.A., Kudryavtsev I.V., Chernikova A.T., Mikhaylova A.A., Aquino A.D., Lagutina D.I., Zaikova E.K., Kalinina O.V., Golovkin A.S. (2022). Effect of Cholecalciferol Supplementation on the Clinical Features and Inflammatory Markers in Hospitalized COVID-19 Patients: A Randomized, Open-Label, Single-Center Study. Nutrients.

[142] De Niet S., Trémège M., Coffiner M., Rousseau A.-F., Calmes D., Frix A.-N., Gester F., Delvaux M., Dive A.-F., Guglielmi E. (2022). Positive Effects of Vitamin D Supplementation in Patients Hospitalized for COVID-19: A Randomized, Double-Blind, Placebo-Controlled Trial. Nutrients.

[143] Torres M., Casado G., Vigón L., Rodríguez-Mora S., Mateos E., Ramos-Martín F., López-Wolf D., Sanz-Moreno J., Ryan-Murua P., Taboada-Martínez M.L. (2022). Changes in the immune response against SARS-CoV-2 in individuals with severe COVID-19 treated with high dose of vitamin D. Biomed. Pharmacother..

[144] Maghbooli Z., Sahraian M.A., Jamalimoghadamsiahkali S., Asadi A., Zarei M.A., Zendehdel A., Varzandi T., Mohammadnabi S., Alijani N., Karimi M. (2021). Treatment with 25-Hydroxyvitamin D3 (Calcifediol) Is Associated with a Reduction in the Blood Neutrophil-to-Lymphocyte Ratio Marker of Disease Severity in Hospitalized Patients with COVID-19: A Pilot Multicenter, Randomized, Placebo-Controlled, Double-Blinded Clinical Trial. Endocr. Pract..

[145] Sabico S., Enani M.A., Sheshah E., Aljohani N.J., Aldisi D.A., Alotaibi N.H., Alshingetti N., Alomar S.Y., Alnaami A.M., Amer O.E. (2021). Effects of a 2-Week 5000 IU versus 1000 IU Vitamin D3 Supplementation on Recovery of Symptoms in Patients with Mild to Moderate COVID-19: A Randomized Clinical Trial. Nutrients.

[146] Villasis-Keever M.A., López-Alarcón M.G., Miranda-Novales G., Zurita-Cruz J.N., Barrada-Vázquez A.S., González-Ibarra J., Martínez-Reyes M., Grajales-Muñiz C., Santacruz-Tinoco C.E., Martínez-Miguel B. (2022). Efficacy and Safety of Vitamin D Supplementation to Prevent COVID-19 in Frontline Healthcare Workers. A Randomized Clinical Trial. Arch. Med. Res..

[147] Caballero-García A., Pérez-Valdecantos D., Guallar P., Caballero-Castillo A., Roche E., Noriega D.C., Córdova A. (2021). Effect of Vitamin D Supplementation on Muscle Status in Old Patients Recovering from COVID-19 Infection. Medicina.

[148] Tan C.W., Ho L.P., Kalimuddin S., Cherng B.P.Z., Teh Y.E., Thien S.Y., Wong H.M., Tern P.J.W., Chandran M., Chay J.W.M. (2020). Cohort study to evaluate the effect of vitamin D, magnesium, and vitamin B12 in combination on progression to severe outcomes in older patients with coronavirus (COVID-19). Nutrition.

[149] Deguchi A. (2015). Curcumin Targets in Inflammation and Cancer. Endocrine Metab. Immune Disord. Drug Targets.

[150] Keihanian F., Saeidinia A., Bagheri R.K., Johnston T.P., Sahebkar A. (2017). Curcumin, hemostasis, thrombosis, and coagulation. J. Cell Physiol..

[151] Pivari F., Mingione A., Brasacchio C., Soldati L. (2019). Curcumin and Type 2 Diabetes Mellitus: Prevention and Treatment. Nutrients.

[152] Dai Q., Zhou D., Xu L., Song X. (2018). Curcumin alleviates rheumatoid arthritis-induced inflammation and synovial hyperplasia by targeting mTOR pathway in rats. Drug Des. Dev. Ther..

[153] Burge K., Gunasekaran A., Eckert J., Chaaban H. (2019). Curcumin and Intestinal Inflammatory Diseases: Molecular Mechanisms of Protection. Int. J. Mol. Sci..

[154] Kahkhaie K.R., Mirhosseini A., Aliabadi A., Mohammadi A., Mousavi M.J., Haftcheshmeh S.M., Sathyapalan T., Sahebkar A. (2019). Curcumin: A modulator of inflammatory signaling pathways in the immune system. Inflammopharmacology.

[155] Liczbiński P., Michałowicz J., Bukowska B. (2020). Molecular mechanism of curcumin action in signaling pathways: Review of the latest research. Phytotherapy Res..

[156] Wang M., Jiang S., Zhou L., Yu F., Ding H., Li P., Zhou M., Wang K. (2019). Potential Mechanisms of Action of Curcumin for Cancer Prevention: Focus on Cellular Signaling Pathways and miRNAs. Int. J. Biol. Sci..

[157] Cohen A.N., Veena M.S., Srivatsan E.S., Wang M.B. (2009). Suppression of Interleukin 6 and 8 Production in Head and Neck Cancer Cells with Curcumin via Inhibition of Iκβ Kinase. Arch. Otolaryngol.-Head Neck Surg..

[158] Ren Y.-X., Ma J.-X., Zhao F., An J.-B., Geng Y.-X., Liu L.-Y. (2018). Effects of Curcumin on Epidermal Growth Factor in Proliferative Vitreoretinopathy. Cell Physiol. Biochem..

[159] Wang L., Li N., Lin D., Zang Y. (2017). Curcumin protects against hepatic ischemia/reperfusion induced injury through inhibiting TLR4/NF-κB pathway. Oncotarget.

[160] He Y.-Q., Zhou C.-C., Yu L.-Y., Wang L., Deng J.-L., Tao Y.-L., Zhang F., Chen W.-S. (2020). Natural product derived phytochemicals in managing acute lung injury by multiple mechanisms. Pharmacol. Res..

[161] Eisenhut M., Shin J.I. (2020). Pathways in the Pathophysiology of Coronavirus 19 Lung Disease Accessible to Prevention and Treatment. Front. Physiol..

[162] Lin C.-Y., Yao C.-A. (2020). Potential Role of Nrf2 Activators with Dual Antiviral and Anti-Inflammatory Properties in the Management of Viral Pneumonia. Infect. Drug Resist..

[163] Anand P., Kunnumakkara A.B., Newman R.A., Aggarwal B.B. (2007). Bioavailability of curcumin: Problems and promises. Mol. Pharm..

[164] Liu W., Zhai Y., Heng X., Che F.Y., Chen W., Sun D., Zhai G. (2016). Oral bioavailability of curcumin: Problems and advancements. J. Drug Target..

[165] Brenna J.T., Salem N., Sinclair A.J., Cunnane S.C. (2009). α-Linolenic acid supplementation and conversion to n-3 long-chain polyunsaturated fatty acids in humans. Prostaglandins Leukot. Essent. Fat. Acids.

[166] Shahidi F., Ambigaipalan P. (2018). Omega-3 Polyunsaturated Fatty Acids and Their Health Benefits. Annu. Rev. Food Sci. Technol..

[167] Guo X.-F., Li K.-L., Li J.-M., Li D. (2019). Effects of EPA and DHA on blood pressure and inflammatory factors: A meta-analysis of randomized controlled trials. Crit. Rev. Food Sci. Nutr..

[168] Malan L., Baumgartner J., Calder P.C., Zimmermann M.B., Smuts C.M. (2014). n–3 Long-chain PUFAs reduce respiratory morbidity caused by iron supplementation in iron-deficient South African schoolchildren: A randomized, double-blind, placebo-controlled intervention. Am. J. Clin. Nutr..

[169] Miles E., Childs C., Calder P. (2021). Long-Chain Polyunsaturated Fatty Acids (LCPUFAs) and the Developing Immune System: A Narrative Review. Nutrients.

[170] Swanson D., Block R., Mousa S.A. (2012). Omega-3 Fatty Acids EPA and DHA: Health Benefits Throughout Life. Adv. Nutr..

[171] Shahbakhti H., Watson R.E.B., Azurdia R.M., Ferreira C.Z., Garmyn M., Rhodes L.E. (2007). Influence of Eicosapentaenoic Acid, an Omega-3 Fatty Acid, on Ultraviolet-B Generation of Prostaglandin-E2 and Proinflammatory Cytokines Interleukin-1β, Tumor Necrosis Factor-α, Interleukin-6 and Interleukin-8 in Human Skin In Vivo¶. Photochem. Photobiol..

[172] Kidd P.M. (2007). Omega-3 DHA and EPA for cognition, behavior, and mood: Clinical findings and structural-functional synergies with cell membrane phospholipids. Altern. Med. Rev..

[173] Gutiérrez S., Svahn S.L., Johansson M.E. (2019). Effects of Omega-3 Fatty Acids on Immune Cells. Int. J. Mol. Sci..

[174] Daak A.A., Elderdery A.Y., Elbashir L.M., Mariniello K., Mills J., Scarlett G., Elbashir M.I., Ghebremeskel K. (2015). Omega 3 (n−3) fatty acids down-regulate nuclear factor-kappa B (NF-κB) gene and blood cell adhesion molecule expression in patients with homozygous sickle cell disease. Blood Cells, Mol. Dis..

[175] Oh D.Y., Talukdar S., Bae E.J., Imamura T., Morinaga H., Fan W.Q., Li P., Lu W.J., Watkins S.M., Olefsky J.M. (2010). GPR120 Is an Omega-3 Fatty Acid Receptor Mediating Potent Anti-inflammatory and Insulin-Sensitizing Effects. Cell.

[176] Duvall M.G., Levy B.D. (2016). DHA- and EPA-derived resolvins, protectins, and maresins in airway inflammation. Eur. J. Pharmacol..

[177] Li Q.-F., Hao H., Tu W.-S., Guo N., Zhou X.-Y. (2020). Maresins: Anti-Inflammatory pro-Resolving Mediators with Therapeutic Potential. Eur Rev Med Pharmacol Sci.

[178] Serhan C.N., Petasis N.A. (2011). Resolvins and Protectins in Inflammation Resolution. Chem. Rev..

[179] Eslamloo K., Xue X., Hall J.R., Smith N.C., Caballero-Solares A., Parrish C.C., Taylor R.G., Rise M.L. (2017). Transcriptome profiling of antiviral immune and dietary fatty acid dependent responses of Atlantic salmon macrophage-like cells. BMC Genom..

[180] Chelstowska S., Widjaja-Adhi M.A.K., Silvaroli J.A., Golczak M. (2016). Molecular Basis for Vitamin A Uptake and Storage in Vertebrates. Nutrients.

[181] Nan W., Si H., Yang Q., Shi H., Zhang T., Shi Q., Li G., Zhang H., Liu H. (2021). Effect of Vitamin A Supplementation on Growth Performance, Serum Biochemical Parameters, Intestinal Immunity Response and Gut Microbiota in American Mink (*Neovison vison*). Animals.

[182] Sirisinha S. (2015). The pleiotropic role of vitamin A in regulating mucosal immunity. Asian Pac. J. Allergy Immunol..

[183] Polcz M.E., Barbul A. (2019). The Role of Vitamin A in Wound Healing. Nutr. Clin. Pract..

[184] Chawla A., Repa J.J., Evans R.M., Mangelsdorf D.J. (2001). Nuclear Receptors and Lipid Physiology: Opening the X-Files. Science.

[185] Penniston K.L., Tanumihardjo S.A. (2003). Vitamin A in dietary supplements and fortified foods: Too much of a good thing?. J. Am. Diet. Assoc..

[186] Timoneda J., Rodríguez-Fernández L., Zaragozá R., Marín M.P., Cabezuelo M.T., Torres L., Viña J.R., Barber T. (2018). Vitamin A deficiency and the lung. Nutrients.

[187] Colt S., Gannon B.M., Finkelstein J.L., Zambrano M.P., Andrade J.K., Centeno-Tablante E., August A., Erickson D., Cárdenas W.B., Mehta S. (2021). Vitamin A status, inflammation adjustment, and immunologic response in the context of acute febrile illness: A pilot cohort study among pediatric patients. Clin. Nutr..

[188] Tepasse P.-R., Vollenberg R., Fobker M., Kabar I., Schmidt H., Meier J., Nowacki T., Hüsing-Kabar A. (2021). Vitamin A Plasma Levels in COVID-19 Patients: A Prospective Multicenter Study and Hypothesis. Nutrients.

[189] Penniston K.L., Tanumihardjo S.A. (2006). The acute and chronic toxic effects of vitamin A. Am. J. Clin. Nutr..

[190] Kouakanou L., Peters C., Brown C.E., Kabelitz D., Wang L.D. (2021). Vitamin C, From Supplement to Treatment: A Re-Emerging Adjunct for Cancer Immunotherapy?. Front. Immunol..

[191] Kennes B., Dumont I., Brohee D., Hubert C., Neve P. (1983). Effect of Vitamin C Supplements on Cell-Mediated Immunity in Old People. Gerontology.

[192] Padayatty S.J., Levine M. (2016). Vitamin C: The known and the unknown and Goldilocks. Oral Dis..

[193] Chaghouri P., Maalouf N., Peters S., Nowak P., Peczek K., Zasowska-Nowak A., Nowicki M. (2021). Two Faces of Vitamin C in Hemodialysis Patients: Relation to Oxidative Stress and Inflammation. Nutrients.

[194] Härtel C., Strunk T., Bucsky P., Schultz C. (2004). Effects of vitamin C on intracytoplasmic cytokine production in human whole blood monocytes and lymphocytes. Cytokine.

[195] Carr A.C., Maggini S. (2017). Vitamin C and Immune Function. Nutrients.

[196] Kim Y., Kim H., Bae S., Choi J., Lim S.Y., Lee N., Kong J.M., Hwang Y.-I., Kang J.S., Lee W.J. (2013). Vitamin C Is an Essential Factor on the Anti-viral Immune Responses through the Production of Interferon-α/β at the Initial Stage of Influenza A Virus (H3N2) Infection. Immune Netw..

[197] Diyya A.S.M., Thomas N.V. (2022). Multiple Micronutrient Supplementation: As a Supportive Therapy in the Treatment of COVID-19. BioMed Res. Int..

[198] Cantorna M.T., Snyder L., Lin Y.-D., Yang L. (2015). Vitamin D and 1,25(OH)2D Regulation of T cells. Nutrients.

[199] Chen S., Sun Y., Agrawal D.K. (2015). Vitamin D deficiency and essential hypertension. J. Am. Soc. Hypertens..

[200] Hu C.-Q., Bo Q.-L., Chu L.-L., Hu Y.-D., Fu L., Wang G.-X., Lu Y., Liu X.-J., Wang H., Xu D.-X. (2020). Vitamin D Deficiency Aggravates Hepatic Oxidative Stress and Inflammation during Chronic Alcohol-Induced Liver Injury in Mice. Oxidative Med. Cell Longev..

[201] Shi S., Feng J., Zhou L., Li Y., Shi H. (2021). Risk Factors for Vitamin D Deficiency in Inflammatory Bowel Disease: A Systematic Review and Meta-Analysis. Turk. J. Gastroenterol..

[202] Yang C.-Y., Leung P.S.C., Adamopoulos I.E., Gershwin M.E. (2013). The Implication of Vitamin D and Autoimmunity: A Comprehensive Review. Clin. Rev. Allergy Immunol..

[203] Chiodini I., Gatti D., Soranna D., Merlotti D., Mingiano C., Fassio A., Adami G., Falchetti A., Eller-Vainicher C., Rossini M. (2021). Vitamin D Status and SARS-CoV-2 Infection and COVID-19 Clinical Outcomes. Front. Public Health.

[204] Chiu S.-K., Tsai K.-W., Wu C.-C., Zheng C.-M., Yang C.-H., Hu W.-C., Hou Y.-C., Lu K.-C., Chao Y.-C. (2021). Putative Role of Vitamin D for COVID-19 Vaccination. Int. J. Mol. Sci..

[205] Lordan R. (2021). Notable Developments for Vitamin D Amid the COVID-19 Pandemic, but Caution Warranted Overall: A Narrative Review. Nutrients.

[206] Rivera D.T., Misra A., Sanil Y., Sabzghabaei N., Safa R., Garcia R.U. (2022). Vitamin D and morbidity in children with Multisystem inflammatory syndrome related to COVID-19. Prog. Pediatr. Cardiol..

[207] Delgado W.D.N.R.A.R.L.D.S.N.T.B., Rouver W.N., Dos Santos R.L. (2020). Protective Effects of Pomegranate in Endothelial Dysfunction. Curr. Pharm. Des..

[208] Esmaeilinezhad Z., Babajafari S., Sohrabi Z., Eskandari M.-H., Amooee S., Boldaji R.B. (2018). Effect of synbiotic pomegranate juice on glycemic, sex hormone profile and anthropometric indices in PCOS: A randomized, triple blind, controlled trial. Nutr. Metab. Cardiovasc. Dis..

[209] Morvaridzadeh M., Sepidarkish M., Daneshzad E., Akbari A., Mobini G.R., Heshmati J. (2019). The effect of pomegranate on oxidative stress parameters: A systematic review and meta-analysis. Complement. Ther. Med..

[210] Sahebkar A., Ferri C., Giorgini P., Bo S., Nachtigal P., Grassi D. (2016). Effects of pomegranate juice on blood pressure: A systematic review and meta-analysis of randomized controlled trials. Pharmacol. Res..

[211] Singh B., Singh J.P., Kaur A., Singh N. (2018). Phenolic compounds as beneficial phytochemicals in pomegranate (*Punica granatum* L.) peel: A review. Food Chem..

[212] Vučić V., Grabež M., Trchounian A., Arsić A. (2019). Composition and Potential Health Benefits of Pomegranate: A Review. Curr. Pharm. Des..

[213] Wu S., Tian L. (2017). Diverse Phytochemicals and Bioactivities in the Ancient Fruit and Modern Functional Food Pomegranate (*Punica granatum*). Molecules.

[214] An X., Zhang Y., Cao Y., Chen J., Qin H., Yang L. (2020). Punicalagin Protects Diabetic Nephropathy by Inhibiting Pyroptosis Based on TXNIP/NLRP3 Pathway. Nutrients.

[215] Cerdá B., Soto C., Albaladejo M.D., Martínez P., Sánchez-Gascón F., Tomás-Barberán F., Espín J.C. (2005). Pomegranate juice supplementation in chronic obstructive pulmonary disease: A 5-week randomized, double-blind, placebo-controlled trial. Eur. J. Clin. Nutr..

[216] de Oliveira J.F.F., Garreto D.V., da Silva M.C.P., Fortes T.S., de Oliveira R.B., Nascimento F.R.F., Da Costa F.B., Grisotto M.A.G., Nicolete R. (2013). Therapeutic potential of biodegradable microparticles containing *Punica granatum* L. (pomegranate) in murine model of asthma. Agents Actions.

[217] Khan N., Afaq F., Kweon M.-H., Kim K., Mukhtar H. (2007). Oral Consumption of Pomegranate Fruit Extract Inhibits Growth and Progression of Primary Lung Tumors in Mice. Cancer Res..

[218] Magrone T., Russo M.A., Jirillo E. (2017). Cigarette Smoke-mediated Perturbations of the Immune Response: A New Therapeutic Approach with Natural Compounds. Endocrine, Metab. Immune Disord.-Drug Targets.

[219] Wang W., Bai J., Zhang W., Ge G., Wang Q., Liang X., Li N., Gu Y., Li M., Xu W. (2020). Protective Effects of Punicalagin on Osteoporosis by Inhibiting Osteoclastogenesis and Inflammation via the NF-κB and MAPK Pathways. Front. Pharmacol..

[220] Sundararajan A., Ganapathy R., Huan L., Dunlap J.R., Webby R.J., Kotwal G.J., Sangster M.Y. (2010). Influenza virus variation in susceptibility to inactivation by pomegranate polyphenols is determined by envelope glycoproteins. Antivir. Res..

[221] Neurath A.R., Strick N., Li Y.-Y., Debnath A.K. (2004). Punica granatum (Pomegranate) juice provides an HIV-1 entry inhibitor and candidate topical microbicide. BMC Infect. Dis..

